# HIF-1 promotes murine breast cancer brain metastasis by increasing production of integrin **β**_3_–containing extracellular vesicles

**DOI:** 10.1172/JCI190470

**Published:** 2025-07-15

**Authors:** Yongkang Yang, Chelsey Chen, Yajing Lyu, Olesia Gololobova, Xin Guo, Tina Yi-Ting Huang, Vijay Ramu, Varen Talwar, Elizabeth E. Wicks, Shaima Salman, Daiana Drehmer, Dominic Dordai, Qiaozhu Zuo, Kenneth W. Witwer, Kathleen L. Gabrielson, Gregg L. Semenza

**Affiliations:** 1 Armstrong Oxygen Biology Research Center and Vascular Program, Institute for Cell Engineering, Johns Hopkins University School of Medicine, Baltimore, Maryland, USA.; 2 Sidney Kimmel Comprehensive Cancer Center at Johns Hopkins, Baltimore, Maryland, USA.; 3 Department of Genetic Medicine and; 4 Department of Molecular and Comparative Pathobiology, Johns Hopkins University School of Medicine, Baltimore, Maryland, USA.; 5 Johns Hopkins University, Baltimore, Maryland, USA.

**Keywords:** Oncology, Vascular biology, Breast cancer, Hypoxia

## Abstract

Brain metastasis is a major cause of breast cancer (BC) mortality, but the cellular and molecular mechanisms have not been fully elucidated. BC cells must breach the blood-brain barrier in order to colonize the brain. Here, we determined that integrin β_3_ (ITGB3) expression mediated by hypoxia-inducible factor 1 (HIF-1) plays a critical role in metastasis of BC cells to the brain. Hypoxia stimulated BC cell migration and invasion ex vivo and brain colonization in vivo. Knockdown of either HIF-1α or ITGB3 expression impaired brain colonization by human or mouse BC cells injected into the cardiac left ventricle. Exposure of BC cells to hypoxia increased expression of ITGB3 and its incorporation into small extracellular vesicles (EVs). EVs harvested from the conditioned medium of hypoxic BC cells showed increased retention in the brain after intracardiac injection that was HIF-1α and ITGB3 dependent. EVs from hypoxic BC cells showed binding to brain endothelial cells (ECs), leading to increased EC–BC cell interaction, increased vascular endothelial growth factor receptor 2 signaling, increased EC permeability, and increased transendothelial migration of BC cells. Taken together, our studies implicate HIF-1–stimulated production of ITGB3^+^ EVs as a key mechanism by which hypoxia promotes BC brain metastasis.

## Abstract 2

Hypoxia-inducible factor 1 activates transcription of the gene coding for integrin beta3, which enables breast cancer cells to metastasize to the brain.

## Graphical abstract

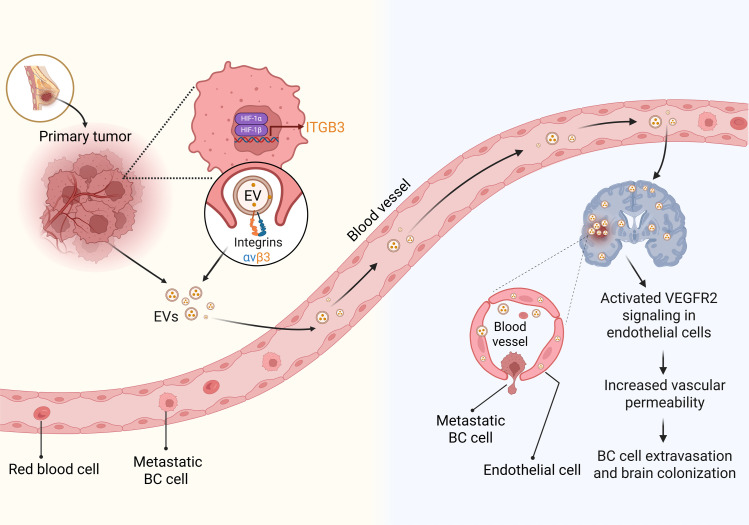

## Introduction

Breast cancer (BC) is one of the most prevalent cancers, affecting millions of women worldwide (1). Metastasis from the initial site of tumor growth to distant sites, especially bone, liver, lung, and brain, is responsible for more than 90% of BC-related deaths, yet metastasis research projects comprised less than 5% of applications to the National Cancer Institute in 2022 (2). Among all patients with metastatic BC, 10%–16% have clinically detected brain metastases, whereas an incidence of 20%–40% has been documented in autopsy studies (3, 4). Among the four major metastatic sites, BC metastasis to the brain is the leading cause of death (5). In the case of HER2^+^ BC and triple-negative BC (TNBC), brain metastasis accounts for more than one-third of metastatic BC (6), and the underlying mechanisms are incompletely understood (7, 8). Less than 2% of BC patients with brain metastasis survive more than 2 years (4). Brain metastasis occurs later in the disease course than metastasis to bone, liver, or lungs, suggesting that metastatic BC cells must overcome additional challenges in order to colonize the brain. A unique obstacle is the blood-brain barrier (BBB), which consists of endothelial cells (ECs) with tight junctions, pericytes, astrocytes, and two basement membranes, which all must be breached for the brain to be colonized (4, 9). Increased permeability of the BBB plays a critical role in this process (5, 10–15), but the underlying molecular and cellular mechanisms have not been fully elucidated.

Key research advances have included the in vivo selection of BC cells with increased tropism to the brain (14, 16–18), the identification of specific genes and signaling pathways that promote (15–17, 19–22) or suppress (23) brain metastasis, and the emerging role of tumor-derived extracellular vesicles (EVs) in promoting metastasis to specific organs (20, 21, 24). EVs, which include exosomes and ectosomes, contain proteins, lipids, RNA, and DNA that are derived from cancer or stromal cells in the primary tumor (25) and are found in the plasma of many patients (26). Among the membrane proteins expressed on the surface of EVs are integrins, heterodimeric transmembrane adhesion proteins with known roles in BC migration and invasion (27) that also promote organotropic metastasis: EVs containing integrins α_6_β_4_ and α_6_β_1_ promote BC metastasis to the lungs by binding to pulmonary fibroblasts and epithelial cells, respectively, whereas EVs containing α_v_β_5_ promote metastasis of pancreatic cancer to the liver by binding to Kupffer cells (20). EVs from brain-metastatic BC cells have been reported to bind to vascular ECs in the brain (20).

Regions of severe intratumoral hypoxia (*P*O_2_ ≤ 10 mmHg = 1.5% O_2_) are present in many advanced BCs (28, 29). Hypoxia-inducible factors (HIFs) are heterodimeric transcriptional activators composed of an O_2_-regulated HIF-α subunit (HIF-1α, HIF-2α, or HIF-3α) and a constitutively expressed HIF-1β subunit (30, 31) that regulate the expression of thousands of RNAs when BC cells are transferred from high (20%) to low (1%) O_2_ (32–34). HIFs bind to hypoxia response elements containing the consensus binding site 5′-(A/G)CGTG-3′ (35) and recruit histone-modifying enzymes to increase transcriptional initiation and elongation (33, 34, 36). Increased HIF-1α expression in tumor biopsies is associated with BC patient mortality, and HIFs activate the expression of gene products that mediate angiogenesis, cancer stem cell specification, epithelial-mesenchymal transition, glucose and lipid metabolism, immune evasion, invasion, and metastasis (37–39). Studies have implicated HIF-1 in BC metastasis to the brain (17, 40), but underlying mechanisms have not been fully elucidated. The goal of the present study is to identify molecular and cellular mechanisms by which HIF-1 promotes BC brain metastasis.

## Results

### Identification of HIF target genes in brain-metastatic BC cells by RNA-Seq and ChIP-Seq.

**Figure 1:**
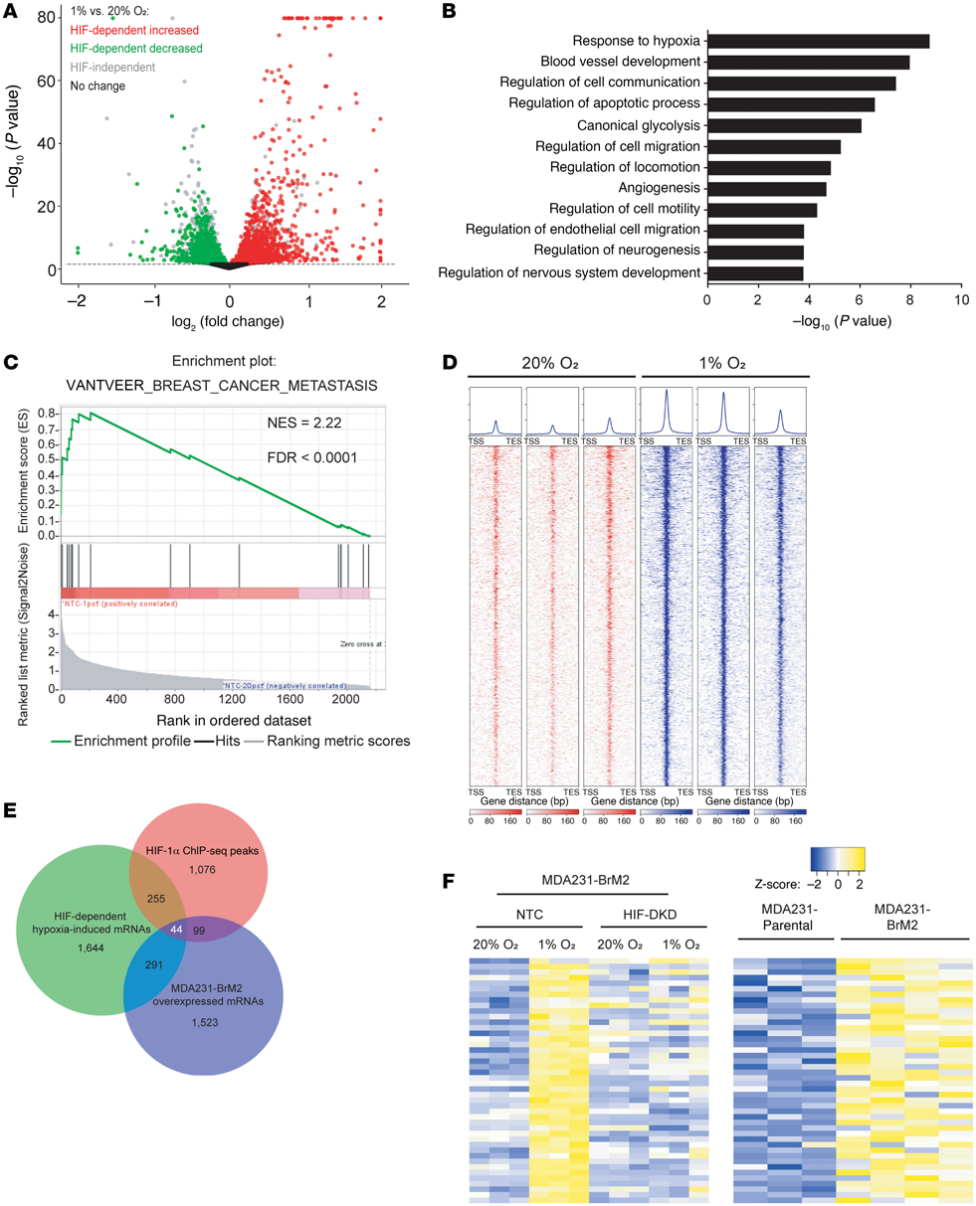
Identification of HIF target genes in brain-metastatic BC cells by RNA-Seq and ChIP-Seq. (**A** and **B**) RNA-Seq analysis of MDA231-BrM2 cells exposed to 20% or 1% O_2_ for 24 hours was performed. Volcano plot (**A**) and Gene Ontology (GO) analysis (**B**) of HIF target genes are shown. (**C**) Gene set enrichment analysis (GSEA) revealed that expression of the “breast cancer metastasis” gene set was significantly correlated with the expression of HIF target genes in MDA231-BrM2 cells. (**D**) HIF-1α binding profiles at significantly called peak summits ± 1 kb in MDA231-BrM2 cells exposed to 20% or 1% O_2_, as determined by ChIP-Seq. (**E**) Venn analysis shows the overlap among 2,234 HIF-dependent hypoxia-induced genes as determined by RNA-Seq; 1,474 genes with HIF-1α binding sites by ChIP-Seq; and 1,957 genes overexpressed in MDA231-BrM2 cells, as compared with MDA231 cells (from ref. 16). (**F**) Heatmaps showing RNA expression of 44 shared genes in NTC versus HIF-double-knockdown cells at 20% or 1% O_2_ (left), and in MDA231 versus MDA231-BrM2 cells at 20% O_2_ (right).

MDA-MB-231-BrM2 (hereafter designated MDA231-BrM2) is a subclone of MDA-MB-231 (hereafter designated MDA231) human TNBC cells in which the capacity for brain metastasis was enriched by in vivo selection (16). Compared with the parental MDA231 cells, 1,957 RNAs were overexpressed in the MDA231-BrM2 subclone (16). To determine whether these overexpressed RNAs were enriched for HIF target genes, we stably transfected MDA231-BrM2 cells with a lentivirus vector encoding a non-targeting control (NTC) short hairpin RNA (shRNA) or vectors encoding shRNAs targeting HIF-1α and HIF-2α (HIF double knockdown [DKD]). These subclones were exposed to 20% or 1% O_2_ for 24 hours, RNA was isolated, and RNA sequencing (RNA-Seq) was performed on 3 biological replicates for each condition. In response to hypoxia, the expression of 2,234 RNAs was significantly increased (FDR < 0.05) in a HIF-dependent manner (Figure 1A). Gene Ontology (GO) analysis revealed that the most highly enriched category was “response to hypoxia” with –log_10_ *P* value > 9 (Figure 1B). HIF-dependent RNAs were also enriched for hypoxia-related pathways (angiogenesis and canonical glycolysis), cancer-related terms (regulation of cell migration), and neural-related terms, suggesting that expression of these RNAs may contribute to brain metastasis (Figure 1B). Consistent with GO analysis, gene set enrichment analysis (GSEA) revealed that HIF-regulated RNAs were highly enriched for the hypoxia, glycolysis, and angiogenesis gene sets (Supplemental Figure 1, A–C; supplemental material available online with this article; https://doi.org/10.1172/JCI190470DS1), as well as a gene set associated with BC metastasis (Figure 1C).

To further investigate the role of HIF-1 in brain metastasis, we performed chromatin immunoprecipitation and DNA sequencing (ChIP-Seq) in MDA231-BrM2 cells exposed to 20% or 1% O_2_ for 16 hours with 3 biological replicates for each condition. Nuclear lysates were immunoprecipitated with HIF-1α antibody, followed by DNA sequencing. This identified 1,474 high-stringency genomic sites at which HIF-1α occupancy was induced by hypoxia (Figure 1D). We then identified RNAs that were (a) overexpressed in MDA231-BrM2 relative to MDA231 cells; (b) induced by hypoxia in MDA231-BrM2 cells in a HIF-dependent manner; and (c) encoded by a gene at which hypoxia-induced HIF-1α binding was identified by ChIP-Seq. We found that 44 RNAs fulfilled all 3 of these criteria (Figure 1, E and F, and Table 1). ITGB3, which encodes integrin subunit β_3_, was of interest because previous studies have implicated integrins α_6_β_1_ and α_6_β_4_ in lung metastasis and α_v_β_5_ in liver metastasis; most importantly, integrin β_3_ was identified in EVs derived from brain-tropic MDA231-BrM2 cells (20), highlighting its potential role in brain metastasis and providing a strong rationale for its further investigation in this context.

### ITGB3 is a direct HIF-1 target gene in brain-metastatic BC cells.

**Figure 2:**
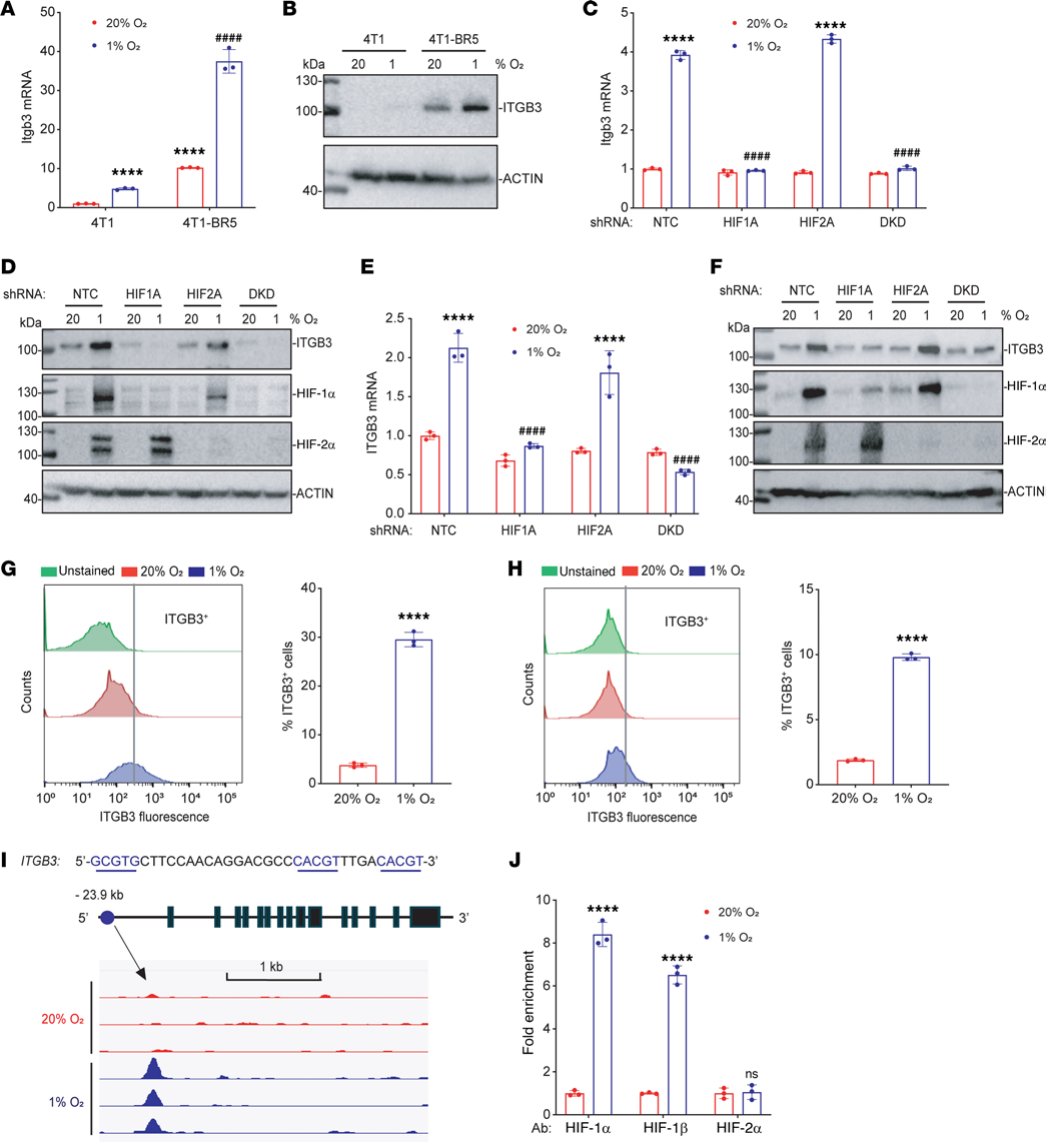
*ITGB3* is a direct HIF-1 target gene. (**A** and **B**) ITGB3 mRNA and protein were analyzed by RT-qPCR (**A**) and immunoblot assays (**B**) in 4T1 and 4T1-BR5 cells exposed to 20% or 1% O_2_. ITGB3 mRNA was quantified relative to 18S rRNA and normalized to mean for 4T1 cells at 20% O_2_; mean ± SD (*n* = 3). ****P < 0.0001 vs. 4T1 at 20% O_2_; ^####^*P* < 0.0001 vs. 4T1 at 1% O_2_ (unpaired 2-tailed Student’s *t* test). (**C**–**F**) 4T1-BR5 (**C** and **D**) or MDA231-BrM2 (**E** and **F**) subclones were exposed to 20% or 1% O_2_, and ITGB3 mRNA or protein levels were analyzed by RT-qPCR (**C** and **E**) and immunoblot assays (**D** and **F**); mean ± SD (*n* = 3). ****P < 0.0001 vs. NTC at 20% O_2_; ^####^*P* < 0.0001 vs. NTC at 1% O_2_ (2-way ANOVA with Tukey’s multiple-comparison test). (**G** and **H**) Flow cytometry histograms showing anti-ITGB3 antibody binding to 4T1-BR5 (**G**) or MDA231-BrM2 (**H**) cells that were exposed to 20% or 1% O_2_; mean ± SD (*n* = 3). ****P < 0.0001 vs. 20% O_2_. (**I**) ChIP-Seq analysis revealed 3 matches to the HIF consensus binding site 5′-(A/G)CGTG-3′ or its complement (underlined) under the HIF-1α peak in the *ITGB3* gene in MDA231-BrM2 cells using the Integrative Genomics Viewer (IGV) genome browser. (**J**) MDA231-BrM2 cells were exposed to 20% or 1% O_2_, and ChIP-qPCR was performed using antibodies against HIF-1α, HIF-1β, or HIF-2α. Primers flanking the nucleotide sequence shown in **I** were used for qPCR, and results were normalized to the mean result at 20% O_2_; mean ± SD (*n* = 3). ****P < 0.0001 vs. 20% O_2_; ns, not significant (unpaired 2-tailed Student’s *t* test).

To further investigate whether ITGB3 expression is O_2_ regulated, we exposed 4T1 mouse TNBC cells and the in vivo–selected brain-metastatic 4T1-BR5 subclone (19) to 20% or 1% O_2_ for 24 or 48 hours, then harvested the cells for reverse transcription (RT) and quantitative real-time PCR (qPCR) (Figure 2A) or immunoblot (Figure 2B) assays, respectively. These results revealed that both ITGB3 mRNA and protein levels were induced by hypoxia. ITGB3 mRNA and protein expression was much higher in 4T1-BR5 than in 4T1 cells (Figure 2, A and B). To determine the HIF-dependence of ITGB3 expression, we established 4T1-BR5 and MDA231-BrM2 subclones stably transduced with a lentiviral expression vector encoding NTC shRNA or shRNA targeting HIF-1α, HIF-2α, or both (double knockdown [DKD]). RT-qPCR and immunoblot assays revealed that knockdown of HIF-1α, but not HIF-2α, abrogated the induction of ITGB3 mRNA and protein expression in both 4T1-BR5 (Figure 2, C and D) and MDA231-BrM2 (Figure 2, E and F) cells exposed to 1% O_2_. Flow cytometry revealed that hypoxia also induced ITGB3 protein expression in the plasma membrane in 4T1-BR5 (Figure 2G) and MDA231-BrM2 (Figure 2H) cells.

Analysis of the ChIP-Seq data from hypoxic MDA231-BrM2 cells revealed a HIF-1 binding peak that was located 24 kb upstream of the human *ITGB3* gene transcription start site, and inspection of the DNA sequence at this site revealed 3 matches to the HIF-1 binding sequence 5′-(A/G)CGTG-3′ (Figure 2I). To confirm that HIF-1 specifically binds at this position, MDA231-BrM2 cells exposed to 20% or 1% O_2_ for 16 hours were analyzed by ChIP-qPCR. Chromatin fragments were precipitated using antibodies against HIF-1α, HIF-1β, or HIF-2α, and fragments containing the putative binding site were quantified by qPCR. Binding of HIF-1α and HIF-1β, but not HIF-2α, was significantly enriched at the *ITGB3* –24 kb site in hypoxic cells (Figure 2J), which is consistent with the finding that hypoxia-induced ITGB3 expression in MDA231-BrM2 cells required HIF-1α, but not HIF-2α (Figure 2, E and F). Taken together, the data presented in Figure 2 demonstrate that *ITGB3* is a hypoxia-inducible HIF-1 target gene in human and mouse TNBC cells that are capable of metastasis to the brain.

### ITGB3 expression is required for hypoxia-induced migration and invasion of brain-metastatic BC cells.

**Figure 3:**
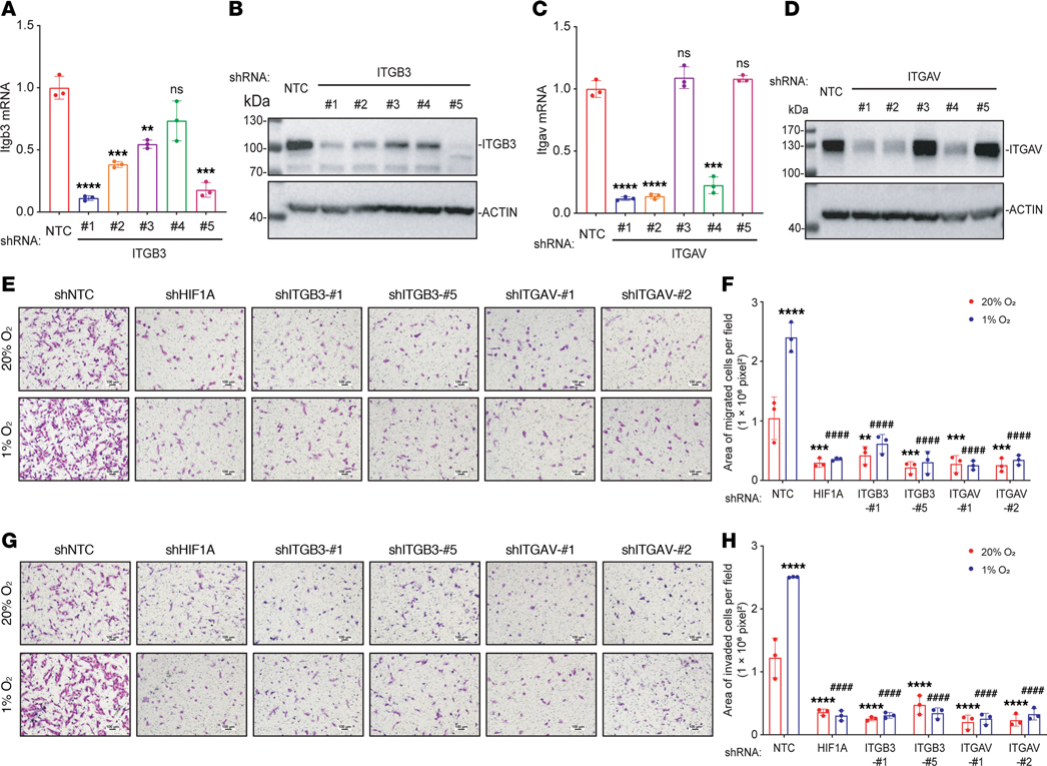
ITGB3 expression is required for hypoxia-induced migration and invasion of brain-metastatic BC cells. (**A**–**D**) 4T1-BR5 subclones that were stably transduced with a lentivirus encoding an NTC shRNA or shRNA targeting ITGB3 or ITGAV were subjected to RT-qPCR (**A** and **C**) or immunoblot assays (**B** and **D**). Data are shown as mean ± SD (*n* = 3). **P < 0.01, ***P < 0.001, ****P < 0.0001 vs. shNTC; ns, not significant vs. shNTC (unpaired 2-tailed Student’s *t* test). (**E**–**H**) 4T1-BR5 subclones were seeded on top of uncoated (**E** and **F**) or Matrigel-coated (**G** and **H**) Boyden chamber inserts and incubated at 20% or 1% O_2_ for 16 (**E** and **F**) or 24 (**G** and **H**) hours. Cells on the underside of the insert were stained with crystal violet and imaged by light microscopy (**E** and **G**; scale bars: 100 μm). The stained area was quantified using ImageJ (NIH) and expressed as mean ± SD (*n* = 3). **P < 0.01, ***P < 0.001, ****P < 0.0001 vs. shNTC at 20% O_2_; ^####^*P* < 0.0001 vs. shNTC at 1% O_2_ (2-way ANOVA with Tukey’s multiple-comparison test).

ITGB3 forms an α_v_β_3_ heterodimer with ITGAV, which has been implicated in cancer cell adhesion, migration, and invasion (41). To explore the biological function of ITGB3 and ITGAV in brain-metastatic BC cells, we established knockdown subclones of 4T1-BR5 and MDA231-BrM2 by transducing them with vectors encoding shRNA targeting ITGB3 or ITGAV. Knockdown efficiency in 4T1-BR5 (Figure 3, A–D) and MDA231-BrM2 cells (Supplemental Figure 2, A–D) was determined by RT-qPCR and immunoblot assays. The effect of ITGB3 or ITGAV shRNA on cell migration and invasion was analyzed using Boyden chamber assays, in which cells were seeded onto a porous membrane that was either uncoated or coated with Matrigel, a sarcoma-derived basement membrane preparation (42). Hypoxia increased the migration and invasion of 4T1-BR5 (Figure 3, E–H) and MDA231-BrM2 (Supplemental Figure 2, E–H) NTC subclones. However, hypoxia-induced migration and invasion were markedly impaired by expression of shRNAs targeting ITGB3, ITGAV, or HIF-1α (Figure 3, E–H, and Supplemental Figure 2, E–H).

**Figure 4:**
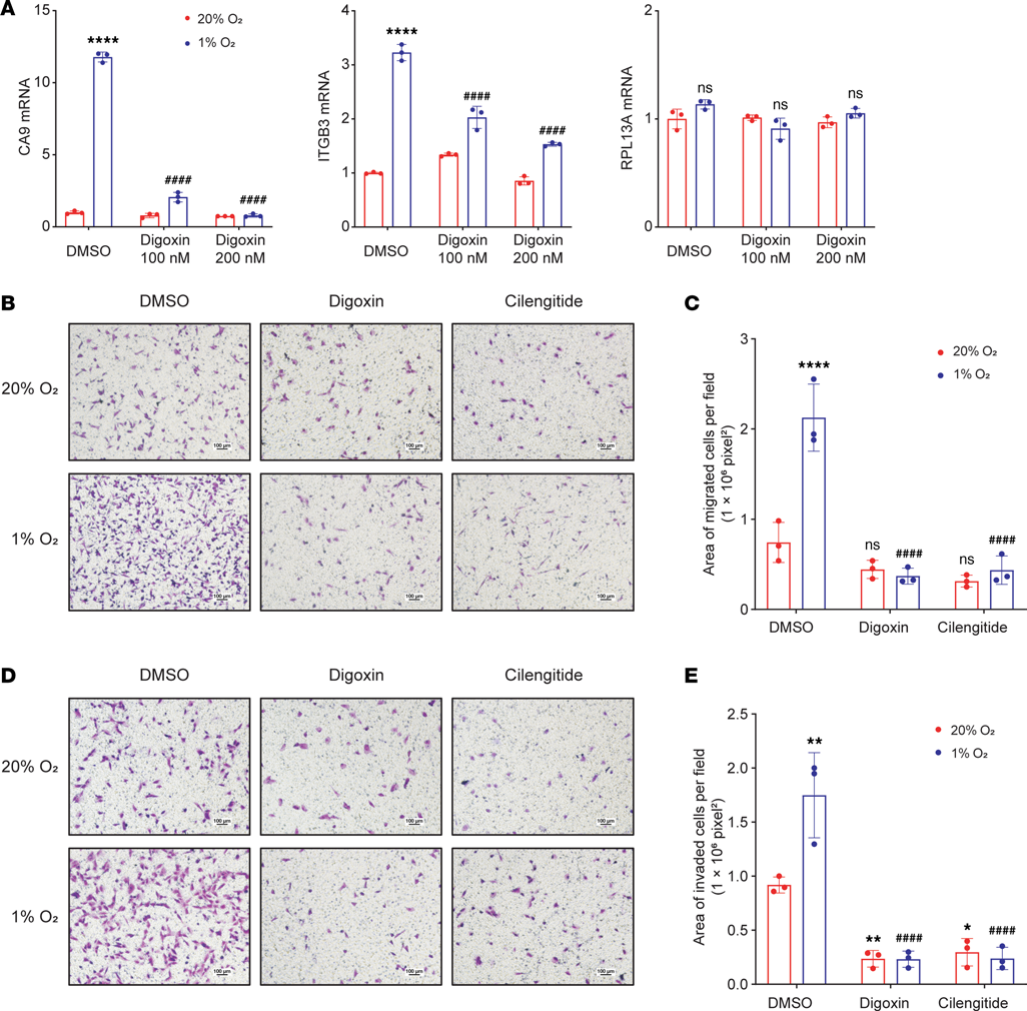
Pharmacological inhibition of HIF-1 or ITGB3 impairs migration and invasion of brain-metastatic BC cells. (**A**) MDA231-BrM2 cells were exposed to 20% or 1% O_2_ for 24 hours in the presence of vehicle (DMSO) or digoxin, and expression of CA9, ITGB3, and RPL13A mRNA was assayed by RT-qPCR. Data are shown as mean ± SD (*n* = 3). ****P < 0.0001 vs. DMSO at 20% O_2_; ^####^*P* < 0.0001 vs. DMSO at 1% O_2_; ns, not significant (2-way ANOVA with Tukey’s multiple-comparison test). (**B**–**E**) MDA231-BrM2 cells were seeded on top of uncoated (**B** and **C**) or Matrigel-coated (**D** and **E**) Boyden chamber inserts and incubated at 20% or 1% O_2_ in the presence of DMSO, digoxin (200 nM), or cilengitide (5 μM). Cells on the underside of the insert were stained with crystal violet and imaged by light microscopy (**B** and **D**; scale bars: 100 μm). The stained area was quantified using ImageJ and expressed as mean ± SD (*n* = 3). *P < 0.05, **P < 0.01, ****P < 0.0001 vs. 20% O_2_ DMSO; ^####^*P* < 0.0001 vs. 1% O_2_ DMSO; ns, not significant (2-way ANOVA with Tukey’s multiple-comparison test).

Digoxin is a HIF inhibitor that blocks HIF-1α protein accumulation and inhibits primary tumor growth, as well as lymph node and lung metastasis, of MDA231 cells implanted in the mammary fat pad of immunodeficient mice (43, 44). MDA231-BrM2 cells were treated with digoxin at 100 or 200 nM during exposure to 20% or 1% O_2_ for 24 hours. RT-qPCR assays revealed that digoxin treatment blocked hypoxia-induced expression of CA9 mRNA, an established HIF-1 target gene product (45), and ITGB3 mRNA, but had no effect on the expression of RPL13A mRNA, which is neither hypoxia-induced nor HIF-regulated (Figure 4A). Cilengitide is an RGD pentapeptide that specifically blocks the binding of integrin α_v_β_3_ to its ligands (46). To further investigate the effect of HIF-1–mediated ITGB3 expression on the migration and invasion of brain-metastatic BC cells, we pharmacologically inhibited HIF-1 or ITGB3 by treating cells with digoxin or cilengitide, respectively. Boyden chamber assays revealed that treatment with digoxin or cilengitide significantly decreased hypoxia-induced migration (Figure 4, B and C) and invasion (Figure 4, D and E) of MDA231-BrM2 cells. Both genetic and pharmacological inhibition of ITGB3 decreased the migration and invasion of MDA231-BrM2 cells at 20% O_2_ (Supplemental Figure 2, E–H, and Figure 4, B–E), indicating that ITGB3 expression is required for basal as well as hypoxia-induced migration and invasion of MDA231-BrM2 cells.

Next, MDA231-BrM2 cells were stably transfected with an ITGB3 expression vector, and ITGB3 protein overexpression was confirmed by immunoblot assays (Supplemental Figure 3A). ITGB3 overexpression significantly increased migration and invasion of MDA231-BrM2 cells under non-hypoxic conditions (Supplemental Figure 3, B–E). Collectively, these results indicate that HIF-1–mediated ITGB3 expression is necessary for increased migration and invasion under hypoxic conditions, and that increased ITGB3 expression is sufficient to increase migration and invasion under non-hypoxic conditions.

### HIF-1–induced ITGB3 expression is required for brain colonization by BC cells.

When hypoxic BC cells enter the circulation, they are no longer subjected to hypoxia. In order to investigate how long hypoxic BC cells maintain increased ITGB3 expression after reoxygenation, we exposed 4T1-BR5 cells to 20% or 1% O_2_ for 48 hours and reoxygenated them for different periods of time. Immunoblot assays revealed that hypoxia-induced ITGB3 expression was maintained elevated for 24 hours and slowly decayed between 48 and 96 hours of reoxygenation (Supplemental Figure 4A). BC cells subjected to hypoxia for 48 hours and reoxygenated for 48 hours maintained an increased capacity for migration and invasion compared with cells maintained at 20% O_2_ (Supplemental Figure 4, B and C). These results suggest that there is a significant window of time in which post-hypoxic BC cells are endowed with increased capacity for migration and invasion.

**Figure 5:**
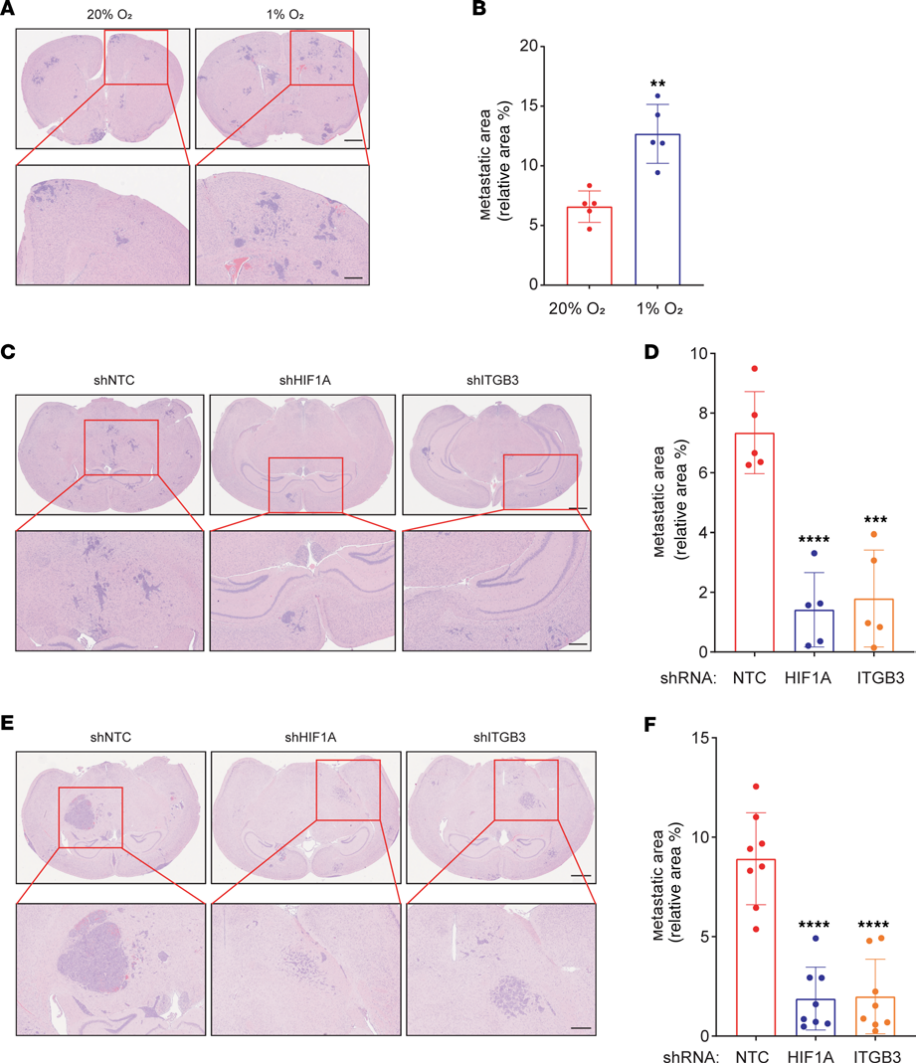
HIF-1–induced ITGB3 expression is required for colonization of brain by BC cells. (**A** and **B**) 4T1-BR5 cells were exposed to 20% or 1% O_2_ for 48 hours and injected into left ventricle of BALB/c mice. On day 14, brains were harvested, and sections were analyzed by H&E staining. Representative images (**A**; scale bars: 200 and 50 μm in top and bottom panels, respectively) and quantification of metastatic area (**B**; mean ± SD; *n* = 5 mice, 3 sections per brain) are shown. **P < 0.01 vs. 20% O_2_ (unpaired 2-tailed Student’s *t* test). (**C** and **D**) Representative images of H&E-stained brain sections (**C**; scale bars: 200 and 50 μm in top and bottom panels, respectively) and quantification of metastatic area (**D**; mean ± SD; *n* = 5 mice, 3 sections per brain) 14 days after intracardiac injection of 4T1-BR5 subclones expressing the indicated shRNA are shown. ***P < 0.001, ****P < 0.0001 vs. shNTC (1-way ANOVA with Tukey’s multiple-comparison test). (**E** and **F**) Representative images of H&E-stained brain sections (**E**; scale bars: 200 and 50 μm in top and bottom panels, respectively) and quantification of metastatic area (**F**; mean ± SD; *n* = 8 mice, 3 sections per brain) 40 days after intracardiac injection of MDA231-BrM2 subclones expressing the indicated shRNA are shown. ****P < 0.0001 vs. shNTC (1-way ANOVA with Tukey’s multiple-comparison test).

To determine whether the hypoxia-induced increases in migration and invasion in vitro were associated with enhanced brain colonization in vivo, we exposed 4T1-BR5 cells to 20% or 1% O_2_ for 48 hours, injected 50,000 cells into the cardiac left ventricle (LV) of syngeneic BALB/c mice, and harvested the brains 14 days later for histological analysis using hematoxylin and eosin (H&E) staining. Prior exposure of 4T1-BR5 cells to hypoxia significantly increased the area of brain colonization (Figure 5, A and B). To further validate the role of HIF-1 and ITGB3 in promoting brain metastasis, we injected the shNTC, shHIF1A, or shITGB3 subclone of 4T1-BR5 (50,000 cells) or MDA231-BrM2 (250,000 cells) into the LV and analyzed brain colonization 14 or 40 days later, respectively. Knockdown of either HIF-1α or ITGB3 markedly decreased brain colonization following LV injection of 4T1-BR5 (Figure 5, C and D) or MDA231-BrM2 (Figure 5, E and F) cells.

### ITGB3 is exported from BC cells via EVs.

**Figure 6:**
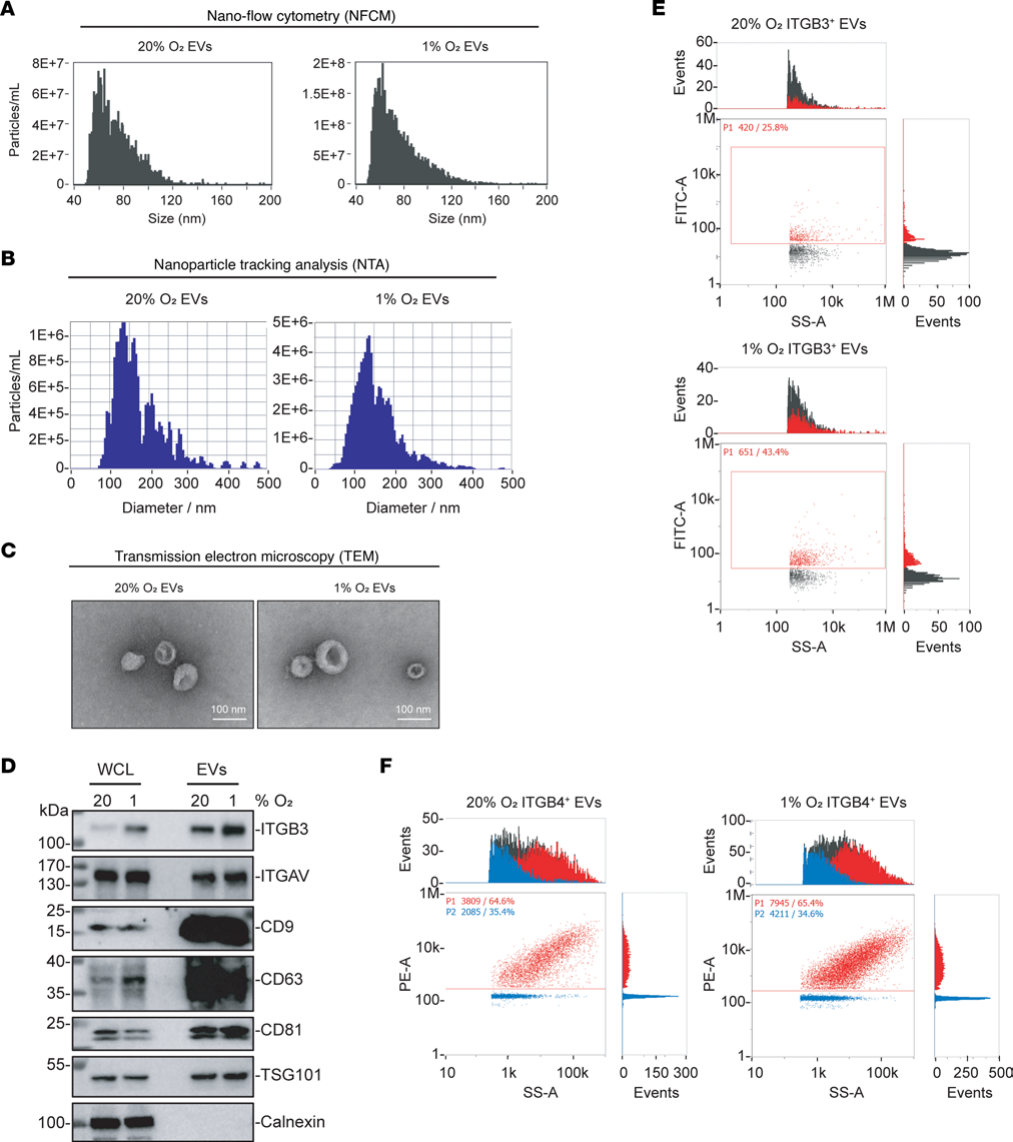
ITGB3 is exported from BC cells via EVs. (**A** and **B**) MDA231-BrM2 cells were exposed to 20% or 1% O_2_ for 48 hours, and EVs were isolated and characterized by nano–flow cytometry (**A**) and nanoparticle tracking analysis (**B**). (**C**) Representative transmission electron microscopy images of EVs derived from MDA231-BrM2 cells are shown. Scale bars: 100 nm. (**D**) EVs and corresponding whole-cell lysates were characterized by immunoblot assays. (**E** and **F**) EVs derived from MDA231-BrM2 cells were stained with antibody against ITGB3 (**E**) or ITGB4 (**F**) and analyzed by nano–flow cytometry. Blue, unstained; red, antibody-stained; black, total particle population.

BC-derived EVs have been shown to remodel the microenvironment of distant organs to promote metastatic niche formation (20) and brain metastasis (21). ITGB3 was identified not only in EVs isolated from brain-tropic MDA231-BrM2 cells, but also in EVs from liver-tropic and lung-tropic BC cells, as well as EVs from liver-tropic and lung-tropic colorectal, gastric, and pancreatic cancer cells (20). To confirm the expression of ITGB3 in EVs and the effect of hypoxia on EV biogenesis, MDA231-BrM2 and 4T1-BR5 cells were exposed to 20% or 1% O_2_ for 48 hours. The conditioned medium was collected, and EVs were isolated by ultracentrifugation. The number and size distribution of EVs were characterized by nano–flow cytometry (NFCM) (Figure 6A and Supplemental Figure 5A) and nanoparticle tracking analysis (NTA) (Figure 6B and Supplemental Figure 5B). Consistent with previous findings (47, 48), both MDA231-BrM2 and 4T1-BR5 cells generated more EVs at 1% O_2_ than at 20% O_2_, but with similar size distribution. Transmission electron microscopy revealed that the EVs had a typical cup-shaped morphology with a size range of 50–150 nm (Figure 6C and Supplemental Figure 5C), which was consistent with the data from NFCM (Figure 6A and Supplemental Figure 5A) and NTA (Figure 6B and Supplemental Figure 5B).

Immunoblot assays of MDA231-BrM2 EVs, as compared with whole-cell lysates, revealed enrichment of ITGB3 and ITGAV, along with the well-established EV markers CD9, CD63, CD81, and TSG101, whereas the EVs did not contain calnexin, a protein localized to endoplasmic reticulum (Figure 6D). Knockdown of ITGAV reduced the incorporation of ITGB3, but not other proteins, in the EVs, and knockdown of ITGB3 reduced ITGAV incorporation into EVs (Supplemental Figure 5G). These results indicate that the observed effects of ITGB3 are likely to occur in the context of α_v_β_3_ heterodimers. Knockdown of both HIF-1α and ITGB3 had no additional effects on EV composition (Supplemental Figure 5, D–G). Hypoxia increased the percentage of EVs containing ITGB3 on their surface from 25.8% to 43.4% in MDA231-BrM2 (Figure 6E) and from 13.8% to 18.6% in 4T1-BR5 (Supplemental Figure 5H), as determined by NFCM. In contrast, there was no effect of hypoxia on the percentage of EVs that displayed ITGB4 (Figure 6F), which was previously identified as a lung metastasis–specific integrin (20). These data indicate that hypoxia-induced ITGB3 is selectively expressed on the surface of EVs produced by brain-tropic MDA231-BrM2 and 4T1-BR5 BC cells.

### HIF-1α expression and ITGB3 expression promote interaction of EVs with brain ECs.

**Figure 7:**
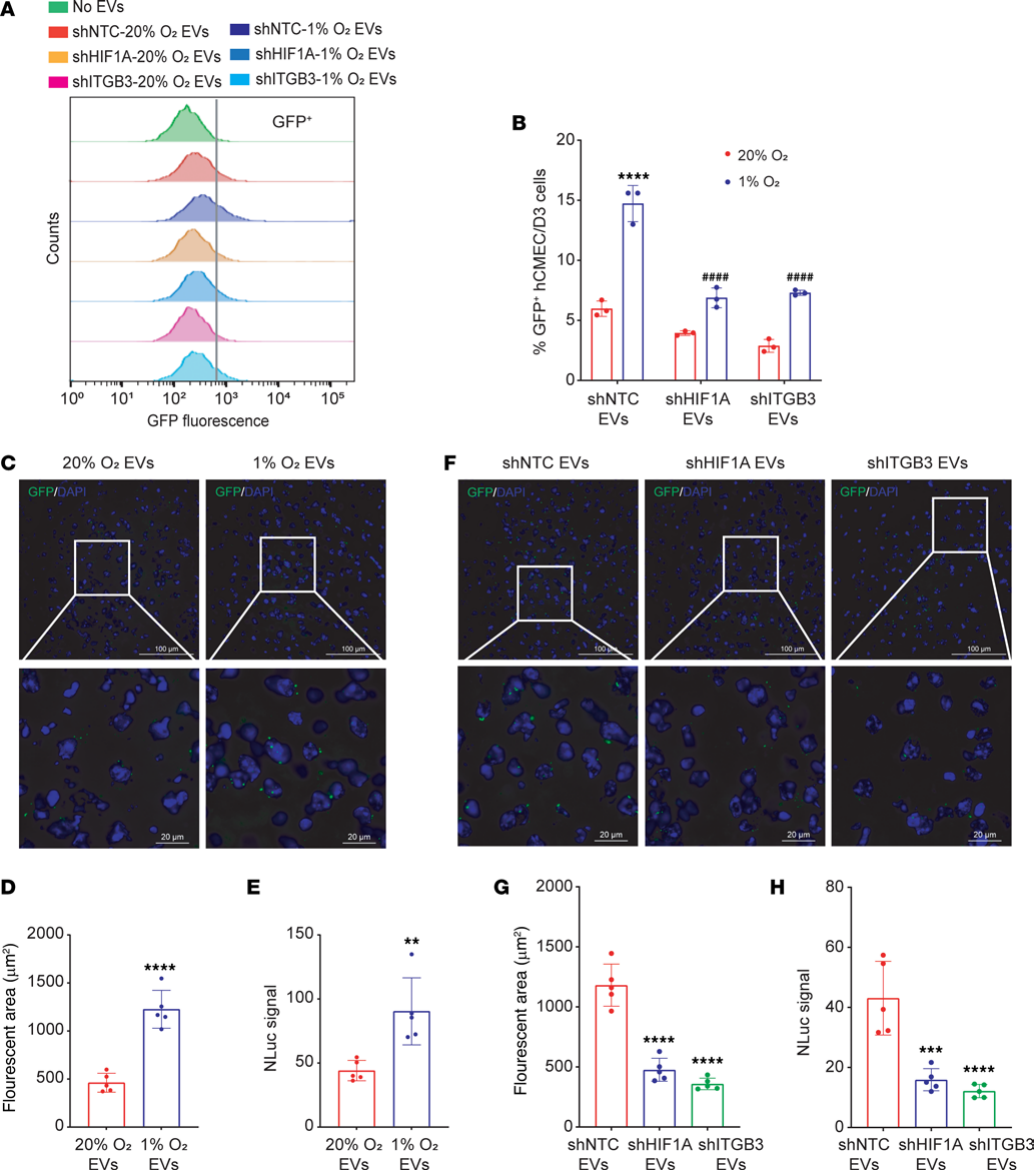
HIF-1α expression and ITGB3 expression promote the interaction of EVs with brain ECs. (**A** and **B**) GFP^+^ PalmGRET-EVs (2 μg; derived from MDA231-BrM2 cells exposed to 20% or 1% O_2_ for 48 hours) were incubated with hCMEC/D3 ECs for 24 hours, and then the cells were analyzed by flow cytometry (**A**) and quantified (**B**; mean ± SD, *n* = 3). ****P < 0.0001 vs. NTC at 20% O_2_; ^####^*P* < 0.0001 vs. NTC at 1% O_2_ (2-way ANOVA with Tukey’s multiple-comparison test). (**C**–**E**) PalmGRET-EVs (10 μg; derived from MDA231-BrM2 cells exposed to 20% or 1% O_2_ for 48 hours) were injected into the left ventricle of BALB/c mice, and then the brains were analyzed by fluorescence microscopy and nanoluciferase assay. Representative fluorescence microscopy images (**C**; scale bars: 100 and 20 μm in top and bottom panels, respectively), quantification of fluorescent area (**D**), and nanoluciferase activity (**E**) are shown; mean ± SD (*n* = 5 mice, 3 sections per brain). **P < 0.01, ****P < 0.0001 vs. 20% O_2_ (unpaired 2-tailed Student’s *t* test). (**F**–**H**) PalmGRET-EVs (10 μg; derived from MDA231-BrM2 subclones exposed to 1% O_2_ for 48 hours) were injected into the left ventricle of BALB/c mice, and then the brains were analyzed by fluorescence microscopy and nanoluciferase assay. Representative fluorescence microscopy images (**F**; scale bars: 100 and 20 μm in top and bottom panels, respectively), quantification of fluorescent area (**G**), and nanoluciferase activity (**H**) are shown; mean ± SD (*n* = 5 mice, 3 sections per brain). ***P < 0.001, ****P < 0.0001 vs. shNTC (1-way ANOVA with Tukey’s multiple-comparison test).

The BBB is a unique semipermeable structure of the microvasculature in the central nervous system that is mainly composed of ECs, pericytes, and astrocytes and can tightly control the translocation of molecules, proteins, and particles to the brain (49). Tumor-derived EVs have been shown to cross the BBB (50). To determine whether EVs produced by brain-tropic BC cells can interact with brain ECs, we generated MDA231-BrM2 subclones expressing the bioluminescence resonance energy transfer reporter protein palmitoylated-EGFP-nanoluciferase (PalmGRET), which can robustly and specifically label the inner membrane of EVs (51). EVs labeled with PalmGRET were isolated from shNTC, shHIF1A, and shITGB3 subclones of MDA231-BrM2 cells that were exposed to 20% or 1% O_2_. Human hCMEC/D3 cells, which are derived from brain ECs and manifest BBB characteristics (52), were incubated with these labeled EVs for 24 hours, followed by flow cytometry to identify ECs with bound PalmGRET^+^ EVs. EVs derived from hypoxic NTC cells showed a significant increase in association with brain ECs; moreover, the association of brain ECs with EVs from shHIF1A and shITGB3 cells was significantly decreased in comparison with EVs from shNTC cells (Figure 7, A and B). Similar results were obtained using HBEC-5i cells (Supplemental Figure 6, A and B), which are also microvascular ECs derived from human brain (53).

To further confirm the interaction of EVs with brain ECs, MDA231-BrM2 cells were exposed to 20% or 1% O_2_ for 48 hours, and PalmGRET^+^ EVs were isolated and injected into the LV. Four hours after injection, we perfused the mice with 4% paraformaldehyde and harvested the brains. Fluorescence microscopy of brain sections revealed a significant increase in the number of PalmGRET^+^ EVs from cells exposed to 1% as compared with 20% O_2_ (Figure 7, C and D). Similarly, nanoluciferase assays of brain lysates showed significantly increased signal from mice injected with EVs from hypoxic MDA231-BrM2 cells (Figure 7E). Next, the shNTC, shHIF1A, and shITGB3 subclones of MDA231-BrM2 cells were exposed to 1% O_2_, PalmGRET^+^ EVs were collected and injected into the LV, and brains were harvested for analysis. Fluorescence microscopy identified a significantly decreased number of EVs from shHIF1A and shITGB3 as compared with shNTC cells (Figure 7, F and G). Similar results were obtained from nanoluciferase assays of brain lysates (Figure 7H). Taken together, the results presented in Figures 6 and 7 demonstrate that hypoxia induces HIF-1–dependent ITGB3 expression in EVs, which is required for the increased retention of circulating EVs in the brain.

### ITGB3^+^ EVs promote interaction of BC cells with ECs and increase endothelial permeability.

**Figure 8:**
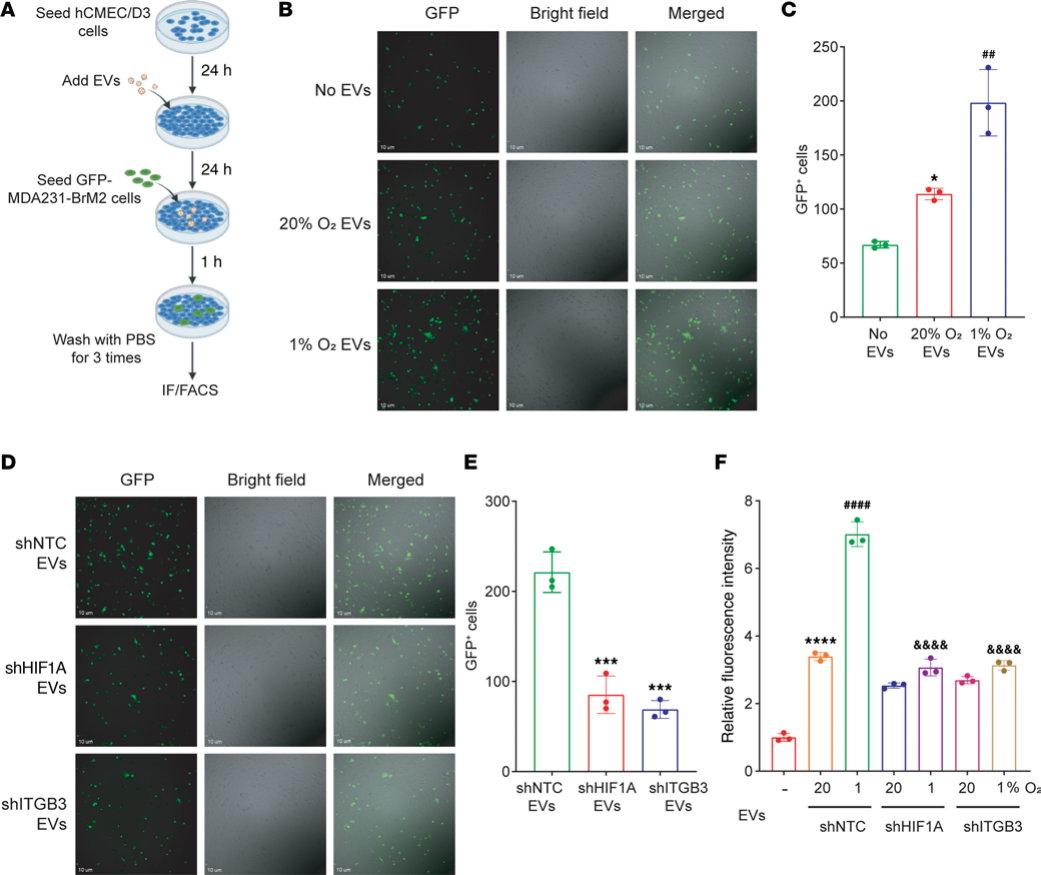
ITGB3^+^ EVs promote interaction of BC cells with ECs and increase endothelial permeability. (**A**–**E**) As shown in the schematic created with BioRender (biorender.com) (**A**), hCMEC/D3 ECs were seeded on 6-well plates, cultured to confluence, and treated for 24 hours with EVs from MDA231-BrM2 cells that were exposed to 20% or 1% O_2_ (**B** and **C**) or subclones that were exposed to 1% O_2_ (**D** and **E**). GFP^+^ MDA231-BrM2 cells were then added onto the hCMEC/D3 monolayer and incubated for 1 hour, and non-adherent cells were removed by washing with PBS. Adherent BC cells were imaged by fluorescence microscopy (**B** and **D**; scale bars: 10 μm) and quantified (**C** and **E**; mean ± SD, *n* = 3). *P < 0.05 or ***P < 0.001 vs. no EVs (**C**) or shNTC EVs (**E**); ^##^*P* < 0.01 vs. 20% O_2_ EVs (**C**) (by 1-way ANOVA with Tukey’s multiple-comparison test). (**F**) hCMEC/D3 ECs were seeded on Boyden chamber filters, cultured to confluence, and incubated with EVs from MDA231-BrM2 subclones for 24 hours. FITC-dextran was added to the upper chamber, and fluorescence in the lower chamber was measured 20 minutes later using a plate reader. Data are shown as mean ± SD (*n* = 3). ****P < 0.0001 vs. no EVs; ^####^*P* < 0.0001 vs. 20% O_2_ shNTC-EVs; ^&&&&^*P* < 0.0001 vs. 1% O_2_ shNTC-EVs (2-way ANOVA followed by Tukey’s multiple-comparison test).

Next, a monolayer of hCMEC/D3 brain ECs was exposed for 24 hours to EVs collected from BC cells cultured at 20% or 1% O_2_. The ECs were then cocultured with GFP-expressing MDA231-BrM2 cells for 1 hour, after which non-adherent cells were removed by gentle rinsing (Figure 8A). Significantly more GFP^+^ MDA231-BrM2 cells interacted with hCMEC/D3 cells when the latter had been pretreated with EVs from BC cells cultured at 1% as compared with 20% O_2_, as determined by immunofluorescence (Figure 8, B and C) or flow cytometry (Supplemental Figure 7, A and B). In addition, EVs from shNTC cells promoted greater interaction of GFP^+^ BC cells with ECs than EVs from shHIF1A or shITGB3 cells, as determined by immunofluorescence (Figure 8, D and E) and flow cytometry (Supplemental Figure 7, C and D).

To investigate the effect of EVs on the permeability of brain ECs, we treated hCMEC/D3 monolayers in Boyden chambers with EVs from shNTC, shHIF1A, and shITGB3 subclones of MDA231-BrM2 cells exposed to 20% or 1% O_2_ for 48 hours. FITC-dextran was added to the upper chamber, and fluorescence in the bottom chamber was measured using a plate reader. EVs from shNTC cells increased EC permeability; EVs from hypoxic shNTC cells had the greatest effect (Figure 8F). The increased permeability induced by EVs from hypoxic shNTC cells was not observed when ECs were treated with EVs from hypoxic shHIF1A or shITGB3 cells (Figure 8F). Taken together, the data in Figure 8 indicate that ITGB3^+^ EVs promote interaction of BC cells with brain ECs and increase endothelial permeability.

### ITGB3^+^ EVs increase permeability of brain ECs by activating VEGFR2 signaling.

**Figure 9:**
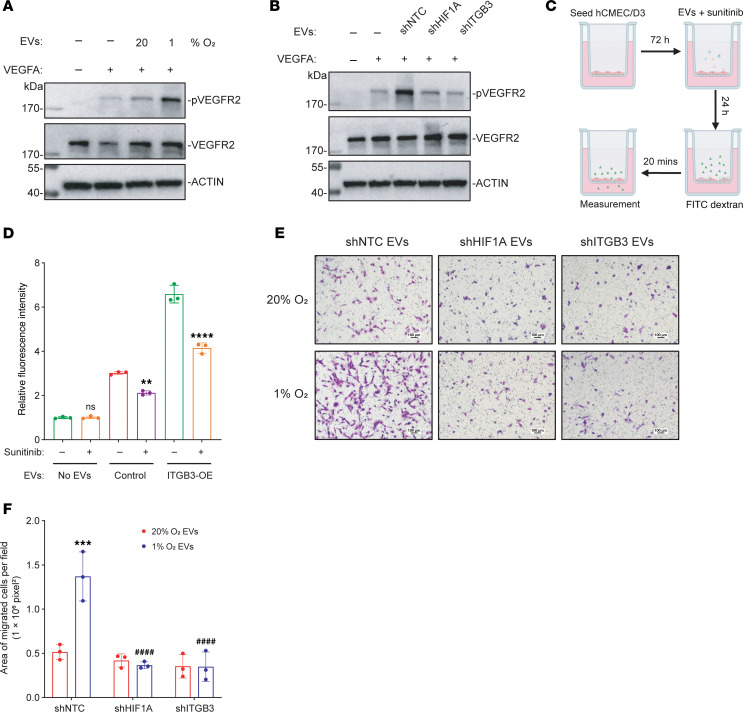
ITGB3^+^ EVs increase permeability of brain ECs by augmenting VEGFR2 signaling. (**A**) hCMEC/D3 cells were incubated for 24 hours with or without EVs (isolated from MDA231-BrM2 cells exposed to 20% or 1% O_2_ for 48 hours), followed by stimulation with VEGFA_165_ for 30 minutes. Whole-cell lysates were prepared for immunoblot assays. (**B**) hCMEC/D3 cells were incubated for 24 hours with or without EVs (isolated from MDA231-BrM2 subclones that were exposed to 1% O_2_ for 48 hours), followed by stimulation with VEGFA_165_ for 30 minutes, and immunoblot assays were performed. (**C** and **D**) hCMEC/D3 cells were seeded into Boyden chamber inserts, cultured to confluence, and incubated with or without EVs from empty vector (Control) or ITGB3-overexpressing (ITGB3-OE) MDA231-BrM2 subclones in the presence of vehicle or 5 μM sunitinib for 24 hours. FITC-dextran was added to the upper chamber, and fluorescence in the lower chamber was measured 20 minutes later and presented as mean ± SD (*n* = 3). ns, not significant vs No EVs without sunitinib; **P < 0.01 vs. control EVs without sunitinib; ****P < 0.0001 vs. ITGB3-OE EVs without sunitinib (2-way ANOVA followed by Tukey’s multiple-comparison test). (**E** and **F**) A monolayer of hCMEC/D3 cells was incubated for 24 hours with EVs (isolated from MDA231-BrM2 subclones that were exposed to 20% or 1% O_2_ for 48 hours), and then MDA231-BrM2 cells were added. BC cells that transmigrated through the EC monolayer to the lower chamber were stained with crystal violet and imaged by light microscopy (**E**; scale bars: 100 μm). The area of stained cells was quantified using ImageJ (**F**; mean ± SD, *n* = 3). ***P < 0.001 vs. 20% O_2_/shNTC-EVs; ^####^*P* < 0.0001 vs. 1% O_2_/shNTC-EVs (2-way ANOVA followed by Tukey’s multiple-comparison test).

Vascular endothelial growth factor (VEGF) receptor 2 (VEGFR2) signaling is the dominant pathway that regulates the permeability of ECs (54), and signaling via integrin α_v_β_3_ has been reported to augment VEGFR2 signaling (55–57). BC cell expression of α_v_β_3_ was also shown to support colonization after intracranial implantation (58). We hypothesized that ITGB3^+^ EVs increase permeability by stimulating VEGFR2 signaling. To test this hypothesis, we exposed hCMEC/D3 brain ECs to EVs isolated from MDA231-BrM2 cells exposed to 20% or 1% O_2_ for 48 hours. After 24 hours, the ECs were treated with recombinant human VEGFA_165_ protein, and cell lysates were prepared. Immunoblot assays revealed that BC cell–derived EVs enhanced VEGFA-induced VEGFR2 phosphorylation/activation, and the effect was augmented when the EVs were derived from hypoxic BC cells (Figure 9A). Furthermore, the augmented VEGFR2 activation by EVs from hypoxic shNTC cells was attenuated when the EVs were isolated from shHIF1A or shITGB3 cells (Figure 9B).

To investigate whether VEGFR2 signaling is implicated in the increased endothelial permeability induced by ITGB3^+^ EVs (Figure 8F), we treated hCMEC/D3 ECs with EVs isolated from control (empty vector) MDA231-BrM2 cells or from MDA231-BrM2 cells overexpressing ITGB3 (ITGB3-OE cells) in the presence or absence of the VEGFR2 inhibitor sunitinib (Figure 9C). Compared with EVs from control cells, EVs from ITGB3-OE cells provoked significantly increased permeability (Figure 9D). Furthermore, coadministration of sunitinib (59) markedly blunted the effect of EVs from ITGB3-OE cells, whereas the effect of sunitinib when coadministered with EVs from control cells was more modest (Figure 9D). These results are consistent with the hypothesis that ITGB3^+^ EVs stimulate augmented VEGFR2 signaling, leading to increased endothelial permeability. This increased permeability should facilitate the transmigration of BC cells through the endothelium. To test this hypothesis, an endothelial monolayer was treated with EVs from shNTC, shHIF1A, or shITGB3 cells exposed to 20% or 1% O_2_, followed by the addition of BC cells to the Boyden chamber. EVs from hypoxic shNTC cells augmented BC cell transmigration across the endothelial monolayer, and this effect was abolished when EVs from hypoxic shHIF1A or shITGB3 cells were used (Figure 9, E and F).

## Discussion

**Figure 10:**
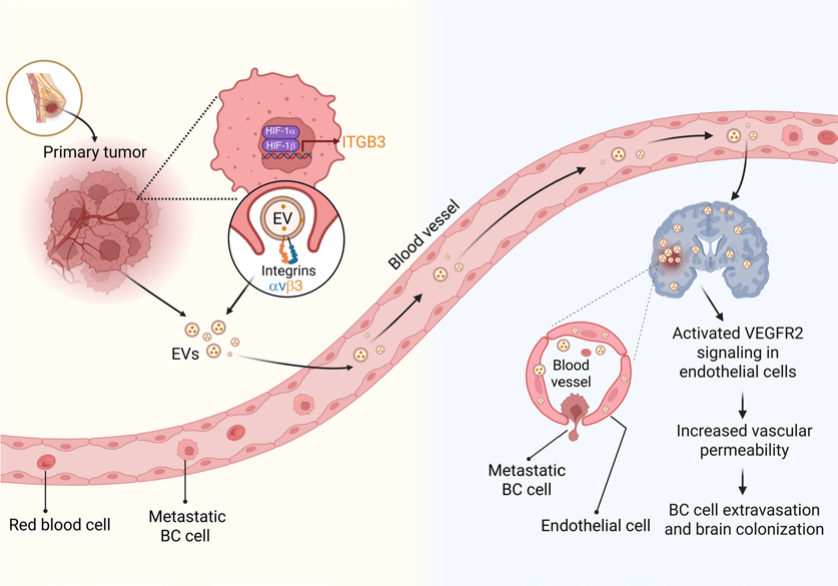
HIF-1–dependent expression of ITGB3 incorporated into EVs promotes BC brain metastasis. Intratumoral hypoxia in primary breast tumors induces the HIF-1–mediated expression of integrin β_3_, which is incorporated into EVs as a heterodimer with integrin α_v_. These EVs are released into the circulation and augment VEGFR2 signaling in brain endothelial cells, which increases vascular permeability, facilitating breaching of the blood-brain barrier and brain colonization by metastatic BC cells. This diagram was created with BioRender (biorender.com).

BC and lung cancer are most commonly associated with brain metastasis, which is thought to be hematogenous in origin (4). Despite its high associated mortality, brain metastasis is understudied, in part because of the absence of a tractable mouse model of metastasis from breast to brain and the considerable technical challenge of safely and accurately injecting BC cells into the LV, which is less than 5 mm in length and beats more than 400 times per minute (60). Compared with bone, liver, or lung metastasis, brain metastasis occurs later in the disease course in BC patients (4), suggesting that, along with the shared challenges of local tissue invasion and vascular intravasation, there are additional obstacles that metastatic BC cells must overcome in order to colonize the brain, which most notably include the BBB. Considerable experimental data indicate that increased BBB permeability plays a critical role in brain metastasis (5, 10–15). Integrins carried by tumor-derived EVs have been shown to promote liver (α_v_β_5_) and lung (α_6_β_4_ and α_6_β_1_) colonization (20). However, the specific integrins involved in brain colonization remained unclear. In this study, we demonstrate (a) increased ITGB3 mRNA and protein expression in hypoxic BC cells that is mediated by HIF-1; (b) increased incorporation of ITGB3 on the surface of EVs derived from hypoxic BC cells; (c) increased short-term uptake in the brain of blood-borne ITGB3^+^ EVs derived from hypoxic BC cells; (d) increased permeability of brain ECs exposed to ITGB3^+^ EVs; (e) increased BC cell transmigration of a brain EC monolayer exposed to ITGB3^+^ EVs; and (f) increased brain colonization by hypoxic BC cells that was HIF-1α and ITGB3 dependent (Figure 10). ITGB3 expression also increased BC invasion of basement membrane in a cell-autonomous manner.

Integrin α_v_β_3_ has been shown to promote tumor angiogenesis and BC growth in the brain after intercranial implantation (58). ITGB3 interacts with VEGFR2 on ECs (56) to augment its tyrosine kinase activity (55). The crosstalk between EV-borne ITGB3 and VEGFR2, leading to increased permeability of brain vascular ECs and increased BC cell transmigration, highlights the critical role of VEGF signaling in BC progression. ITGAV expression was not upregulated by hypoxia, but it was detected in EVs, suggesting that the ability of ITGB3^+^ EVs to stimulate brain metastasis requires integrin α_v_β_3_ heterodimer formation. HIF-dependent production of VEGFA (61) in response to intratumoral hypoxia leads to angiogenesis (62, 63) and increased vascular permeability (64) in the primary tumor, which facilitates the intravasation of BC cells into the circulation (65). VEGFA production by BC cells also plays a major role in immune evasion by stimulating recruitment to the primary tumor of immunosuppressive myeloid-derived suppressor cells, regulatory T cells, and tumor-associated macrophages; by inhibiting the recruitment and activation of cytotoxic T cells; and by inhibiting dendritic cell maturation and activation — effects that are primarily mediated via VEGFR1 (66–68). As we and others (20, 69) have shown for ITGB3, VEGFA has been detected in cancer cell–derived EVs (70). Thus, HIF-1 stimulates production by BC cells of an activator (VEGFA) and an amplifier (ITGB3) of VEGFR2-mediated permeability. Sunitinib has been used to treat renal cell carcinoma patients with brain metastases, demonstrating safety and antitumor activity (71, 72). We showed that blocking VEGFR2 signaling with sunitinib inhibits the ITGB3^+^ EV–mediated increase in endothelial permeability, suggesting that sunitinib or other VEGF pathway inhibitors might be useful for the prevention or treatment of BC brain metastasis.

The current studies do not establish the direct mechanism by which EV-borne ITGB3 engages VEGFR2 signaling. VEGFA in cancer-derived EVs was reported to be inaccessible to anti-VEGFA antibodies and to exert an effect via intracrine signaling in ECs (70). ITGB3 was detected on the surface of BC cell–derived EVs by NFCM (Figure 6E), suggesting that it interacts with VEGFR2 extracellularly. On the other hand, it was previously reported that ITGB3 on EVs promotes their cellular uptake, but the target cells studied were BC cells, not ECs (73). Further studies are required to determine whether ITGB3-VEGFR2 crosstalk is extracellular or intracellular. Since VEGFR2 is ubiquitously expressed by ECs, it is likely that ITGB3 promotes metastasis to both the brain and other organs via this VEGFR2-mediated effect on vascular permeability. In support of this conclusion, ITGB3 knockdown in MDA231 cells has been shown to inhibit bone colonization after saphenous vein injection (73) and lung colonization after tail vein injection (74); intravenous injections of ITGB3 shRNA nanoparticles blocked metastasis of MDA231 cells from breast to lungs (75); ITGB3 knockdown in 4T1 cells inhibited metastasis from breast to bone and lungs (76); and ITGB3 was identified in EVs from liver-tropic and lung-tropic cancer cells (20).

Although we have shown ITGB3 to play a critical role in brain metastasis in human and mouse BC models, it is also important to note that analysis of hypoxia-induced RNA-Seq and HIF-1α ChIP-Seq datasets together with a brain metastasis–selected RNA-Seq dataset (16) from MDA231-BrM2 cells identified, in addition to *ITGB3*, 43 other genes that overlapped all 3 datasets (Table 1). A remarkable number of these genes have been implicated in BC metastasis to bone and/or lungs, including *ADM*, *ANGPTL4*, *BCL11A*, *DUSP1*, *IMP3*, *LOXL2*, *NOL3*, *NREP*, and *SOS1* (77–86), suggesting that they also promote brain metastasis. It is likely that other genes on this list also play important roles, either in the general process of BC metastasis or in brain metastasis–specific processes. For example, LOXL2-enriched EVs mediate hypoxia-induced premetastatic niche formation in the lungs and are associated with poor outcome in head and neck cancer (87). Further studies are required to identify gene products that are required for brain, liver, lung, or bone colonization. MDA231 subclones that have been selected for metastasis to bone (80) or lung (88) have also been generated. However, it can be argued that proteins that promote metastasis to all four sites might be better therapeutic targets. Following this logic upstream, HIF-1 acts as a master regulator that coordinates the expression of a large battery of genes that together powerfully effect a physiological or pathophysiological outcome. Based on previous studies, it is also likely that, in hypoxic BC cells with augmented brain-metastatic capacity, HIF-1 has also programmed augmented cancer stem cell and immune evasive properties (37, 39, 89–92), which together are major determinants of the lethal cancer phenotype.

While our findings provide insight into the role of ITGB3^+^ EVs in BC brain metastasis, this study has limitations that warrant acknowledgment. First, because of technical constraints in our experimental setup, we were unable to demonstrate EV uptake by brain ECs in vivo. However, prior studies demonstrated that EVs derived from brain-tropic cancer cells, including MDA231-BrM2, preferentially associate with brain ECs in vivo (20), aligning with our in vitro observations (Figure 7, A and B). Second, while vascular permeability defects are a critical functional outcome of EV-mediated signaling, reproducing such phenotypes in murine models in prior studies has required repeated intraocular EV injections over an extended period (e.g., 10 doses across 20 days), a protocol that poses significant technical and logistical challenges. Consequently, our study does not establish a direct causal link between EV exposure and vascular leakage in vivo. Future investigations should prioritize optimizing EV tracking methodologies and long-term dosing regimens to validate these mechanisms and their functional implications in the brain microenvironment. The role of astrocytes and pericytes in this process also deserves further investigation.

## Methods

### Sex as a biological variable.

Our study exclusively examined female mice because the vast majority of BC occurs in females.

### Cell culture and reagents.

MDA-MB-231, HEK293T, HBEC-5i, and 4T1 cells were purchased from ATCC. MDA-MB-231-BrM2 (16) and 4T1-BR5 (19) cells were gifts from Joan Massagué (Memorial Sloan Kettering Cancer Center, New York, New York, USA) and Suyun Huang (Virginia Commonwealth University, Richmond, Virginia, USA), respectively. hCMEC/D3 cells (52) were a gift from James Hansen (Yale University, New Haven, Connecticut, USA). MDA-MB-231, MDA231-BrM2, 4T1, 4T1-BR5, and HEK293T cells were maintained in Dulbecco’s modified Eagle medium (DMEM). Culture media were supplemented with 10% (vol/vol) fetal bovine serum (FBS) and 1% (vol/vol) penicillin-streptomycin. HBEC-5i cells were maintained in DMEM:F12 medium supplemented with 10% (vol/vol) FBS, 1% (vol/vol) penicillin-streptomycin, and 40 μg/mL endothelial growth supplement. hCMEC/D3 cells were cultured in EndoGRO-MV Complete Media Kit (Complete Media Kit (MilliporeSigma, catalog SCME004)supplemented with 1 ng/mL fibroblast growth factor-2. Cells were cultured at 37°C in a 5% CO_2_, 95% air incubator. Cells were subjected to hypoxia in a workstation flushed with a mixture of 1% O_2_, 5% CO_2_, and 94% N_2_. Cell lines were mycoplasma free, and human BC cells were authenticated by short tandem repeat DNA profiling analysis by the Johns Hopkins Genetic Resources Core Facility (JHGRCF). Digoxin, cilengitide, and sunitinib were dissolved in DMSO at 1,000× relative to their final concentration in tissue culture media. See Supplemental Table 1 for reagent sources.

### Reverse transcription and qPCR.

Total RNA was extracted from cells using TRIzol reagent, and cDNA synthesis was performed using a High Capacity RNA-to-cDNA kit (ThermoFisher Scientific, catalog 4387406). SYBR Green qPCR Master Mix (Bio-Rad, catalog 1725125) was used for qPCR. The expression of target gene mRNA (E), relative to 18S rRNA, was calculated based on the threshold cycle (Ct) as E = 2^–Δ(ΔCt)^, in which ΔCt = Ct_target_ – Ct_18S_ and Δ(ΔCt) = ΔCt_treatment_ _sample_ – ΔCt_control_ _sample_. PCR primer sequences are shown in Supplemental Table 2.

### RNA-Seq.

MDA231-BrM2 subclones were seeded into 6-well plates in 3 biological replicates and exposed to 20% or 1% O_2_ for 24 hours. Total RNA was isolated using TRIzol and treated with DNase. Library preparation and sequencing using the NovaSeq 6000 platform (Illumina) were performed by the JHGRCF High-Throughput Sequencing Center. RNA-Seq data were processed using Genialis Expressions software as described in Supplemental Methods and submitted to the NCBI’s Gene Expression Omnibus (GEO) database (GSE268804). Data were analyzed and visualized using Heatmapper for heatmaps (93), BioVenn for Venn diagrams (94), and GOnet for GO analysis (95). Gene set enrichment analysis (GSEA) was performed using GSEA version 2.1.0 software (96).

### Immunoblot assay.

Whole-cell lysates were prepared in modified RIPA buffer with protease inhibitor cocktail. Equal amounts of lysate were fractionated by SDS-PAGE and transferred onto nitrocellulose membranes. The blots were incubated with primary antibodies (Supplemental Table 3) overnight at 4°C and with secondary antibodies at room temperature for 1 hour, followed by signal detection using ECL Plus (GE Healthcare, catalog RPN2236).

### ChIP-Seq and data analysis.

MDA-MB-231-BrM2 cells were exposed to 20% or 1% O_2_ for 16 hours, fixed in 1% formaldehyde for 10 minutes, quenched with 0.125 M glycine for 5 minutes, and processed for ChIP (97). ChIP-Seq data were obtained and analyzed as described in Supplemental Methods and submitted to GEO (GSE268802).

### Lentivirus transduction.

The sources for pLKO.1-puro lentiviral shuttle vectors encoding shRNA, pLenti6.3/V5-ITGB3, and pLenti-PalmGRET (51) are shown in Supplemental Table 4. The lentiviral plasmids were cotransfected into HEK293T cells with packaging plasmids psPAX2 and pCMV-VSV-G. Viruses were harvested 48 hours after transfection and passed through a 0.45-μm filter. Puromycin was added to the medium of lentivirus-transduced cells for selection of stable transfectants at a concentration of 0.5 (MDA231-BrM2) or 2 (4T1-BR5) μg/mL.

### ChIP-qPCR assay.

MDA231-BrM2 cells were incubated at 20% or 1% O_2_ for 16 hours, cross-linked in 1% formaldehyde for 10 minutes at 37°C, quenched in 0.125 M glycine for 5 minutes at 37°C, and lysed with SDS lysis buffer. Chromatin was sheared by sonication, and lysates were precleared with salmon sperm DNA/protein A–agarose for 1 hour at 4°C and incubated with antibodies against HIF-1α, HIF-1β, or HIF-2α (Supplemental Table 3) in the presence of protein A–agarose beads overnight. After washes with low-salt, high-salt, LiCl, and Tris-EDTA buffers, DNA was eluted in 1% SDS/0.1 M NaHCO_3_, and cross-links were reversed by addition of 0.2 M NaCl. DNA was purified by phenol-chloroform extraction and ethanol precipitation and analyzed by qPCR. Primer sequences are shown in Supplemental Table 5.

### EV separation and characterization.

EV separation was performed as previously described (98, 99). Briefly, a total of 50 mL of culture-conditioned medium was collected from cells after exposure to 20% or 1% O_2_ for 48 hours. The medium was centrifuged at 1,000*g* for 5 minutes at 4°C to remove dead cells, and 2,000*g* for 10 minutes at 4°C to remove cell debris. Supernatant was centrifuged at 10,000*g* for 30 minutes at 4°C to remove large vesicles and remaining cell debris. Then, supernatant was centrifuged at 100,000*g* for 140 minutes at 4°C using a Beckman Coulter SW32Ti swinging bucket rotor (catalog 369650) and washed with 1× phosphate-buffered saline (PBS; pH 7.4). The 100,000*g* pellets containing small EVs were resuspended in 0.5 mL of 1× PBS, and 50-μL aliquots were stored at –80°C for downstream assays. Characterization of EVs by NFCM, NTA, and transmission electron microscopy is described in Supplemental Methods.

### Flow cytometry.

BC cells were incubated with Fc Block (BD Pharmingen, catalog 564220) followed by fluorophore-conjugated anti-ITGB3 antibody (Supplemental Table 3). Samples were analyzed using a FACSCalibur (BD Biosciences) flow cytometer. Dead cells were gated out by side scatter and forward scatter analysis.

### Transwell assays.

Migration, invasion, endothelial permeability, and transendothelial migration (100) assays were performed as described in Supplemental Methods.

### Adhesion assay.

100,000 HBEC-5i or hCMEC/D3 ECs were seeded on Matrigel-coated 24-well plates, incubated for 16 hours, and incubated with EVs in 1× PBS or PBS alone for 24 hours, followed by addition of 100,000 GFP-MDA231-BrM2 cells. After 1 hour, cells were washed 3 times with cold 1× PBS, and adherent BC cells were scored by fluorescence microscopy or flow cytometry.

### Animal studies.

Ultrasound-guided LV injection was performed as previously described (101, 102). For MDA231-BrM2 subclones, 250,000 cells were injected into 7- to 8-week-old female athymic nude mice (The Jackson Laboratory, strain 002019) in 50 μL of 1× PBS. Mice were euthanized on day 40 after injection. For 4T1-BR5 cells/subclones, 50,000 cells were injected into 7- to 8-week-old female BALB/cJ mice (The Jackson Laboratory, strain 000651) in 50 μL of 1× PBS. Mice were euthanized on day 14. Brains were fixed, paraffin-embedded, sectioned, and stained with H&E at the Johns Hopkins University Oncology Tissue and Imaging Service Core Laboratory. Metastases were quantified as described in Supplemental Methods. PalmGRET-EVs (10 μg) were injected into the LV of female BALB/cJ mice in 50 μL of 1× PBS. Four hours after injection, brains were harvested for nanoluciferase assay or fluorescence microscopy.

### Nanoluciferase assays.

Tissue homogenates were loaded undiluted at 50 μL per well in duplicate as previously described (99). Nano-Glo substrate furimazine (Promega) was diluted 1:50 in assay buffer, 50 μL was added per well, and bioluminescence was measured immediately on a VICTOR Nivo plate reader (PerkinElmer) in bioluminescence mode with an integration time of 20 milliseconds.

### Transcardiac perfusion, fresh-frozen tissue sectioning, and fluorescence microscopy.

Transcardiac perfusion was performed on mice under deep isoflurane anesthesia by insertion of a blunted 27-gauge needle from the LV into the ascending aorta with controlled circulation of 1× PBS at a rate of 7 mL/min using a peristaltic pump (Masterflex). Mice were euthanized, and the brains were harvested. Brains were resected 1 mm posterior to the bregma, and the anterior portions were embedded in Tissue-Tek OCT, snap-frozen in liquid nitrogen, and cryopreserved at –80°C. All brains (bregma –1 to +2 mm) were sliced into 15-μm coronal sections using a Cryostar NX70 Cryostat (Thermo Fisher Scientific). Tissue sections were distributed equally across the brain anterior-posterior axis. Detection of EVs by immunofluorescence is described in Supplemental Methods.

### Statistics.

All data are expressed as mean ± SD. Differences between 2 or multiple groups were analyzed by unpaired, 2-tailed Student’s *t* test or ANOVA, respectively, and *P* values less than 0.05 were considered significant.

### Study approval.

Protocols were approved by the Johns Hopkins University Animal Care and Use Committee and were in accordance with the NIH *Guide for the Care and Use of Laboratory Animals* (National Academies Press, 2011).

### Data availability.

The RNA-Seq and ChIP-Seq data were deposited in the NCBI Gene Expression Omnibus database (GEO GSE268804 and GSE268802). The codes for the quantification of Transwell migration and invasion assays and GFP immunofluorescence are available in GitHub: https://github.com/VarenTalwar/transwell-cellcounting (commit ID: 8b29e8f3bc99c706f157d7dc601e0f626639268a). All other data that support the findings of this study are present in the main paper and supplemental tables and figures. Values for all data points in graphs are reported in the Supporting Data Values file. Raw immunoblot data are reported in the full unedited blot and gel images file.

## Author contributions

YY and GLS conceived and designed research studies and experiments. YY performed most of the experiments and acquired data, with help from CC, YL, VR, VT, EEW, TYTH, QZ, and D Drehmer. CC performed ChIP-Seq and data analyses. YY, CC, and VR performed RNA-Seq and dataset analyses. OG and KWW helped with EV-related experiments. XG and KLG helped with intracardiac injections. TYTH performed frozen sectioning and fluorescence microscopy and analyzed the data. TYTH, EEW, SS, YL, and D Dordai helped with animal experiments. YY and GLS interpreted data and wrote the manuscript. GLS supervised the study. All authors reviewed and approved the manuscript.

## Supplementary Material

Supplemental data

Unedited blot and gel images

Supporting data values

## Figures and Tables

**Table 1:**
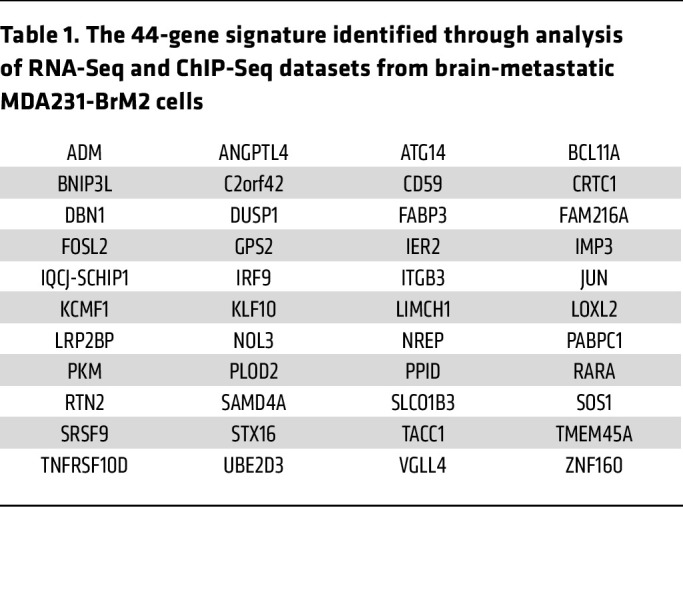
The 44-gene signature identified through analysis of RNA-Seq and ChIP-Seq datasets from brain-metastatic MDA231-BrM2 cells

## Version 1 07/15/2025

Electronic publication
