# Supplementary material for: HIF-1 promotes murine breast cancer brain metastasis by increasing production of integrin β3–containing extracellular vesicles
Source: J Clin Invest. 2025 Jul 15;135(14):e190470. doi: 10.1172/JCI190470 (PMC12259260; doi:10.1172/JCI190470)
Supplement: Supplemental data [file jci-135-190470-s149.pdf]

Supplemental Material

HIF-1 promotes murine breast cancer brain metastasis by increasing production of integrin β3-containing extracellular vesicles

Yongkang Yang[1,2], Chelsey Chen[1,3], Yajing Lyu[1,3], Olesia Gololobova[4], Xin Guo[2,4], Tina Yi-Ting Huang[1,3], Vijay Ramu[5], Varen Talwar[5], Elizabeth E. Wicks[1], Shaima Salman[1,2], Daiana Drehmer[1,3], Dominic Dordai[1,3], Qiaozhu Zuo[1], Kenneth W. Witwer[4], Kathleen L. Gabrielson[2,4], and Gregg L. Semenza[1,2,3]

[1]Armstrong Oxygen Biology Research Center and Vascular Program, Institute for Cell Engineering, Johns Hopkins University School of Medicine, Baltimore, MD 21205, USA. [2]Sidney Kimmel Comprehensive Cancer Center at Johns Hopkins, Baltimore, MD 21231, USA. [3]Department of Genetic Medicine, Johns Hopkins University School of Medicine, Baltimore, MD 21205, USA. [4]Molecular and Comparative Pathobiology, Johns Hopkins University School of Medicine, Baltimore, MD 21205, USA. [5]Johns Hopkins University, Baltimore, MD 21218, USA.

Corresponding Author: Gregg L. Semenza, Miller Research Building, Suite 671, 733 N. Broadway, Baltimore, MD 21205. Fax: 443-287-5618; E-mail: gsemenza@jhmi.edu

Supplemental Tables 1-5

Supplemental Figures 1-7

Supplemental Methods

Supplemental Tables

Supplemental Table 1. Chemical reagents and their commercial suppliers.

Reagent	Supplier	Catalog #
Cilengitide	Selleck Chemicals	S6387
Digoxin	MilliporeSigma	D6003
DMEM:F12 medium	Corning	10-092-CV
Dulbecco's modified Eagle medium (DMEM)	Corning	10-013-CV
ECL Plus	GE Healthcare	RPN2236
EndoGRO™-MV Complete Media Kit	MilliporeSigma	SCME004
Endothelial growth supplement	MilliporeSigma	E2759
Fetal bovine serum	GeminiBio	100-106
Fibroblast growth factor 2	R&D Systems	234-FSE-025/CF
High Capacity RNA-to-cDNA kit	ThermoFisher Scientific	4387406
Human Fc block	BD Pharmingen	564220
Isoflurane	MWI Veterinary	502017
Nano-Glo	Promega	N1110
Penicillin-streptomycin	Sigma-Aldrich	P0781
Protease inhibitor cocktail	MilliporeSigma	P8340
Puromycin	ThermoFisher Scientific	A1113803
RIPA buffer	MilliporeSigma	20-188
Salmon sperm/protein A agarose	MilliporeSigma	16-157
Sunitinib	Selleck Chemicals	S1042
SYBR Green qPCR Master Mix	Bio-Rad	1725125
Tissue-Tek OCT	Sakura Finetek	4583
TRIzol	ThermoFisher Scientific	15596026

Supplemental Table 2. Oligonucleotide sequence of RT-qPCR primers.

Gene	Nucleotide sequence (5' to 3')
Human *ITGB3*	Forward: GTGACCTGAAGGAGAATCTGC
	Reverse: CCGGAGTGCAATCCTCTGG
Human *HIF1A*	Forward: CCACAGGACAGTACAGGATG
	Reverse: TCAAGTCGTGCTGAATAATACC
Human *ITGAV*	Forward: ATCTGTGAGGTCGAAACAGGA
	Reverse: TGGAGCATACTCAACAGTCTTTG
Human *18S rRNA*	Forward: CGGCGACGACCCATTCGAAC
	Reverse: GAATCGAACCCTGATTCCCCGTC
Mouse *Itgb3*	Forward: CCACACGAGGCGTGAACTC
	Reverse: CTTCAGGTTACATCGGGGTGA
Mouse *Itgav*	Forward: CCGTGGACTTCTTCGAGCC
	Reverse: CTGTTGAATCAAACTCAATGGGC
Mouse *18S rRNA*	Forward: GTAACCCGTTGAACCCCATT
	Reverse: CCATCCAATCGGTAGTAGCG

Supplemental Table 3. Antibody information for immunoblot (IB), chromatin immunoprecipitation (ChIP) and flow cytometry (FCM) assays.

**Primary Antibody**	**Source**	**Catalog #**	**Application**	**Usage**
HIF-1α	BD Biosciences	610959	IB	1:500
HIF-1α	Cayman Chemical	10006421	IB	1:500
HIF-1α	Novus Biologicals	NB100-479	ChIP	2 μg
HIF-1β	Novus Biologicals	NB100-110	ChIP	2 μg
HIF-2α	Novus Biologicals	NB100-122	ChIP/IB	2 μg/1:500
ITGB3	Novus Biologicals	AF2266	IB	1:1000
ITGAV	Novus Biologicals	AF1219	IB	1:2000
CD81	Novus Biologicals	NB100-65805	IB	1:500
CD63	Novus Biologicals	NBP2-32830	IB	1:500
CD9	Novus Biologicals	NBP2-67310	IB	1:1000
TSG101	Novus Biologicals	NB200-112	IB	1:1000
Calnexin	Novus Biologicals	NB100-1965	IB	1:2000
ITGB3	Novus Biologicals	FAB2266G (AF488)	FCM	2 μg
ITGB4	Novus Biologicals	FAB4060P (PE)	FCM	2 μg
p-VEGFR2 (Y1214)	Novus Biologicals	AF1766	IB	1:500
VEGFR2	Novus Biologicals	AF357	IB	1:1000
Actin	Santa Cruz Biotechnology	sc-47778	IB	1:5000
**Secondary Antibody**	**Source**	**Catalog #**	**Application**	**Usage**
Mouse IgG HRP	GE Healthcare	NA931V	IB	1:2000
Rabbit IgG HRP	GE Healthcare	NA934V	IB	1:2000
Goat IgG HRP	Santa Cruz Biotechnology	sc-2354	IB	1:2000

Supplemental Table 4. shRNAs in lentiviral vectors and other plasmids used in this study.

shRNA	Source	Identifier
Human HIF1A shRNA	Sigma Aldrich	TRCN0000003810
Human HIF2A shRNA	Sigma Aldrich	TRCN0000003806
Mouse HIF1A shRNA	Sigma Aldrich	TRCN0000232222
Mouse HIF2A shRNA	Sigma Aldrich	TRCN0000428636
Human ITGB3 shRNA-#1	Sigma Aldrich	TRCN0000003235
Human ITGB3 shRNA-#2	Sigma Aldrich	TRCN0000003236
Human ITGB3 shRNA-#3	Sigma Aldrich	TRCN0000003237
Human ITGAV shRNA-#1	Sigma Aldrich	TRCN0000003239
Human ITGAV shRNA-#2	Sigma Aldrich	TRCN0000010768
Human ITGAV shRNA-#3	Sigma Aldrich	TRCN0000010769
Mouse ITGB3 shRNA-#1	Sigma Aldrich	TRCN0000009616
Mouse ITGB3 shRNA-#2	Sigma Aldrich	TRCN0000009617
Mouse ITGB3 shRNA-#3	Sigma Aldrich	TRCN0000009618
Mouse ITGB3 shRNA-#4	Sigma Aldrich	TRCN0000009619
Mouse ITGB3 shRNA-#5	Sigma Aldrich	TRCN0000009620
Mouse ITGAV shRNA-#1	Sigma Aldrich	TRCN0000066588
Mouse ITGAV shRNA-#2	Sigma Aldrich	TRCN0000066589
Mouse ITGAV shRNA-#3	Sigma Aldrich	TRCN0000066591
Mouse ITGAV shRNA-#4	Sigma Aldrich	TRCN0000066592
Mouse ITGAV shRNA-#5	Sigma Aldrich	TRCN0000426214
pLenti6.3/V5-ITGB3	DNASU	HsCD00937082
pLenti-PalmGRET	Addgene	Plasmid #158221

Supplemental Table 5. Nucleotide sequence of ChIP-qPCR primers.

Gene	Nucleotide sequence (5' to 3')
ITGB3 HIF-1 binding site	Forward: TAGGGAAAACGGTGTTGGAG
	Reverse: TGAAGGCAAAGAGACGGACT

Supplemental Figures

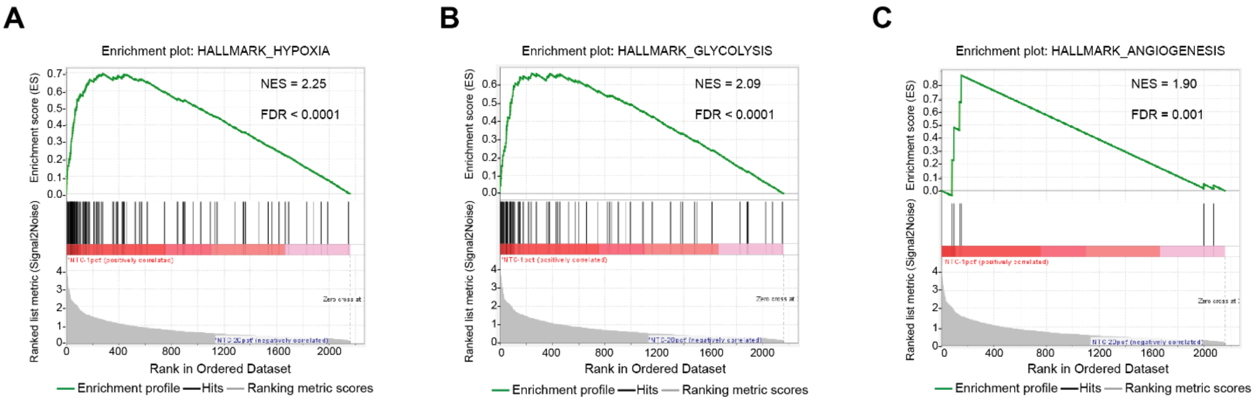

Supplemental Figure 1. Gene set enrichment analysis (GSEA) of HIF target genes in MDA231-BrM2 cells. (**A**-**C**) GSEA revealed that HIF-mediated hypoxia-induced genes are enriched for genes involved in hypoxia (**A**), glycolysis (**B**) and angiogenesis (**C**). Data were analyzed by Genialis using DESeq2 program, two-sided Wald test with multiple test correction, FDR < 0.05.

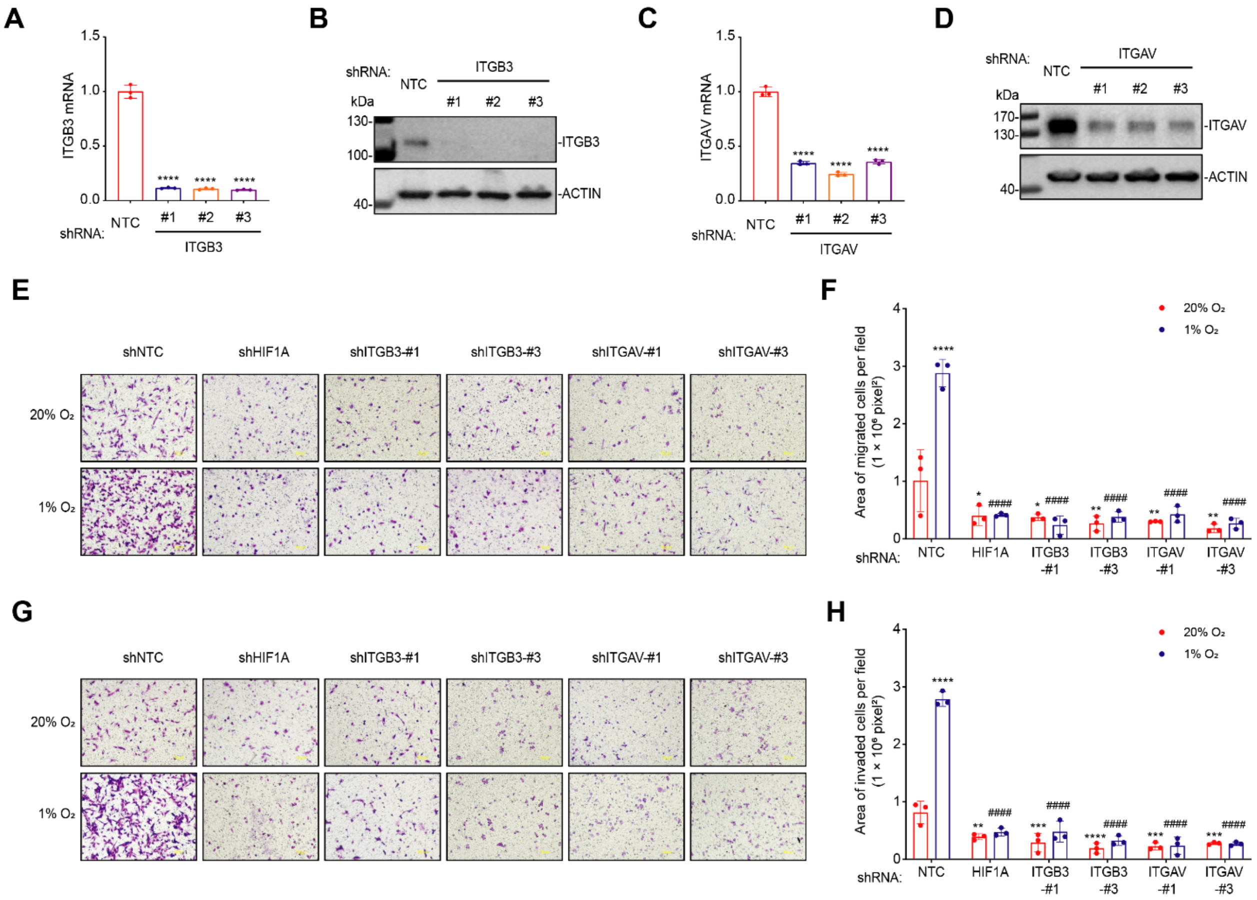

Supplemental Figure 2. ITGB3 expression is required for hypoxia-induced migration and invasion of brain metastatic BC cells. (**A-D**) MDA231-BrM2 subclones were stably transduced with a lentiviral vector encoding a non-targeting control (NTC) short hairpin RNA (shRNA) or shRNA targeting ITGB3 or ITGAV (#1 to #3). Knockdown efficiency was confirmed by RT-qPCR (**A** and **C**) and immunoblot assays (**B** and **D**). Data are shown as the mean ± SD (n = 3). $^{****}P < 0.0001$ versus shNTC (unpaired two-tailed Student's t test). (**E-H**) shNTC, shHIF1A, shITGB3-#1, shITGB3-#3, shITGAV-#1 and shITGAV-#3 subclones of MDA231-BrM2 cells were seeded on top of uncoated (**E** and **F**) or Matrigel-coated (**G** and **H**) Boyden chamber inserts and incubated at 20% or 1% O_2 for 16 (**E** and **F**) or 24 (**G** and **H**) hours. Cells on the underside of the insert were stained with crystal violet and imaged by light microscopy (**E** and **G**; scale bar, 100 μm). The stained area was quantified using ImageJ and expressed as mean ± SD (n = 3). $^{*}P < 0.05$, $^{**}P < 0.01$, $^{***}P < 0.001$, $^{****}P < 0.0001$ versus shNTC at 20% O_2; $^{\#\#\#\#}P < 0.0001$ versus shNTC at 1% O_2 (two-way ANOVA with Tukey's multiple comparisons test).

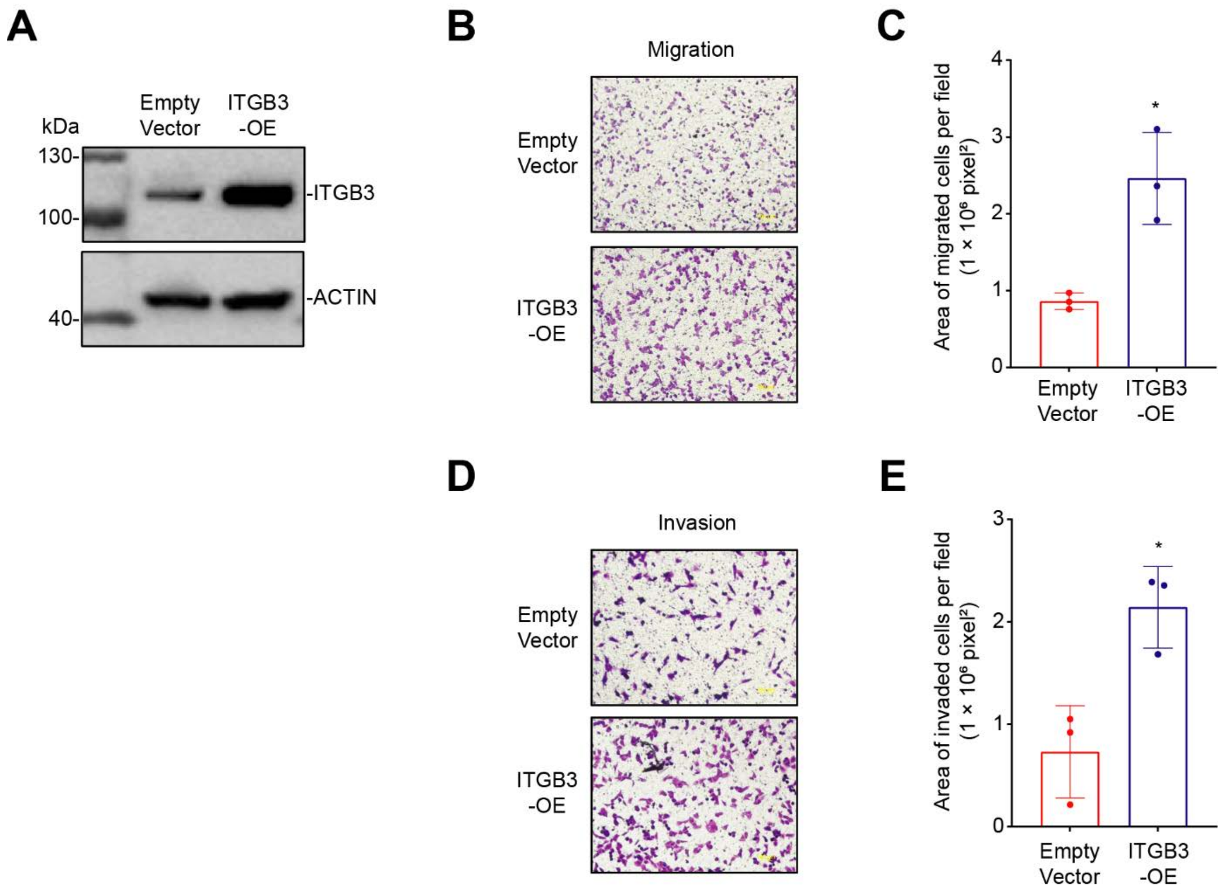

Supplemental Figure 3. ITGB3 overexpression (OE) promotes brain metastatic BC cells migration and invasion. (**A**) Protein levels of ITGB3 were detected by immunoblot assays to confirm the OE efficiency in MDA231-BrM2 cells. (**B-E**) MDA231-BrM2 cells transduced with control empty vector or ITGB3-OE were seeded on top of uncoated (**B**) or Matrigel-coated (**D**) Boyden chamber inserts and incubated at 20% O_2 for 16 (**B** and **C**) or 24 (**D** and **E**) hours. Cells on the underside of the insert were stained with crystal violet and imaged by light microscopy (**B** and **D**; scale bar, 100 μm). The stained area was quantified using ImageJ and expressed as mean ± SD (n = 3). $^{*}P < 0.05$ versus control empty vector (unpaired two-tailed Student's t test).

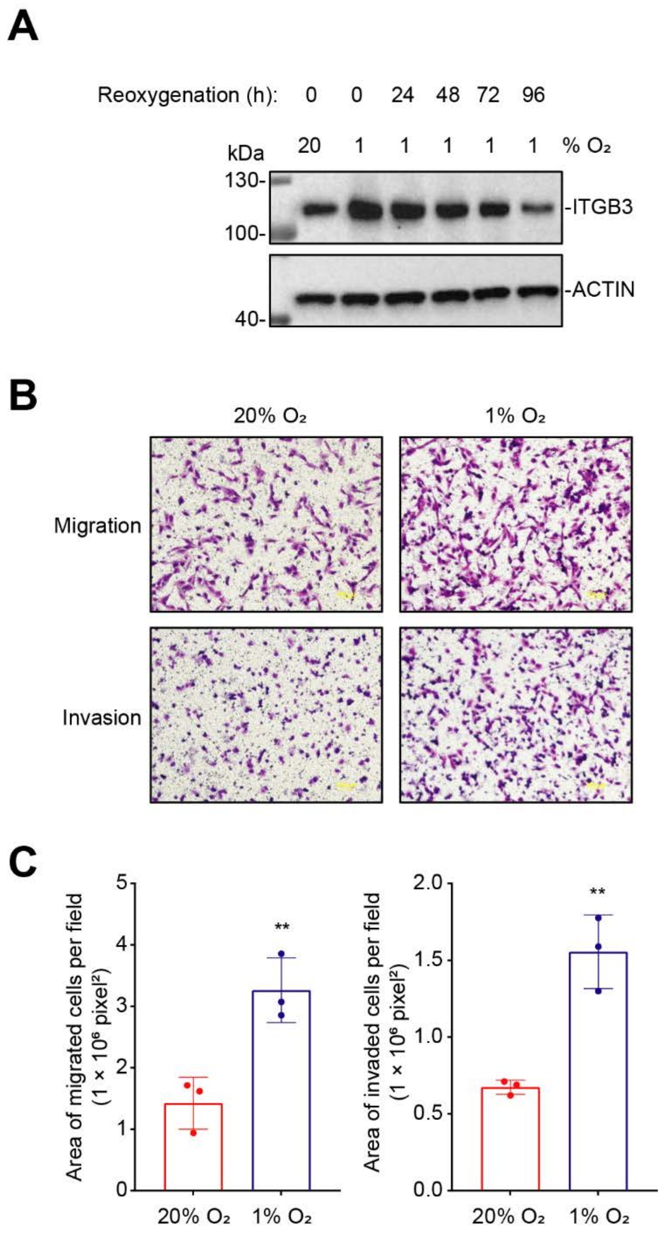

Supplemental Figure 4. Post-hypoxic 4T1-BR5 cells retain an increase in the expression of ITGB3 and the ability of migration and invasion. (**A**) 4T1-BR5 cells were exposed to 20% or 1% O_2 for 48 hours and then reoxygenated for 0, 24, 48, 72 or 96 hours, followed by immunoblot assays. (**B** and **C**) 4T1-BR5 cells that were exposed to 20% or 1% O_2 for 48 hours were seeded on top of uncoated (**B**, upper panels; **C**, left panel) or Matrigel-coated (**B**, bottom panels; **C**, right panel) Boyden chamber inserts and incubated at 20% O_2 for 16 (**B**, upper panels; **C**, left panel) or 24 (**B**, bottom panels; **C**, right panel) hours. Cells on the underside of the insert were stained with crystal violet and imaged by light microscopy (**B**; scale bar, 100 μm). The stained area was quantified using ImageJ and expressed as mean ± SD ($n = 3$). $^{**}P < 0.01$ versus 20% O_2 (unpaired two-tailed Student's t test).

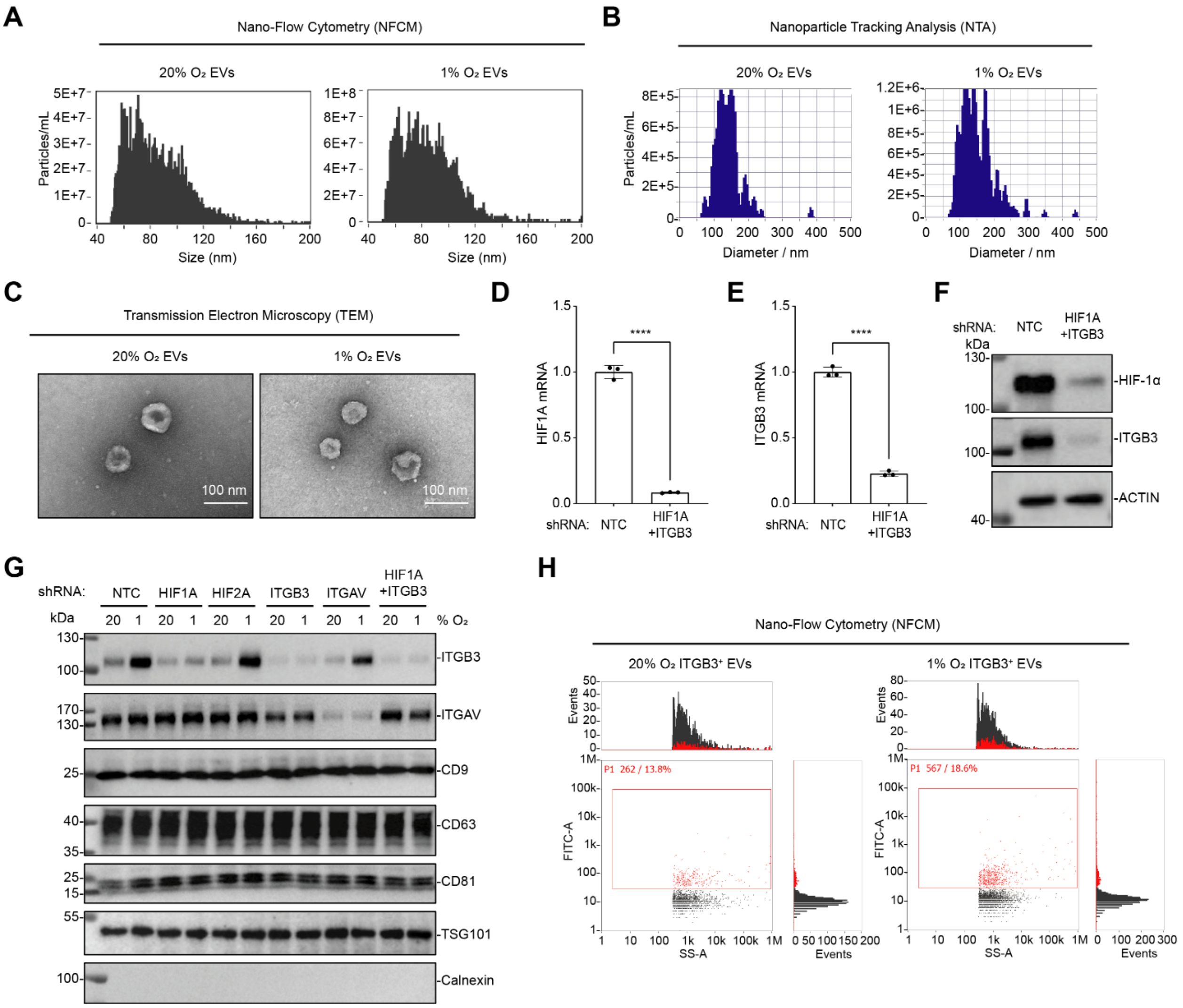

Supplemental Figure 5. ITGB3 is exported from BC cells via extracellular vesicles (EVs). (**A** and **B**) 4T1-BR5 cells were exposed to 20% or 1% O_2 for 48 hours and EVs were isolated and characterized by Nano-flow cytometry (NFCM, **A**) and Nanoparticle Tracking Analysis (NTA, **B**). (**C**) Representative transmission electron microscopic (TEM) images of EVs derived from 4T1-BR5 cells. (**D-E**) Subclones of MDA231-BrM2 cells were stably transduced with a lentivirus encoding a non-targeting control (NTC) short hairpin RNA (shRNA) or shRNAs targeting HIF1A and ITGB3. HIF1A (**D**) and ITGB3 (**E**) mRNA were quantified by RT-qPCR and normalized to NTC. Data are shown as mean ± SD (n = 3). $^{****}P < 0.0001$ versus shNTC (unpaired two-tailed Student's t test). (**F**) MDA231-BrM2 subclones were exposed to 20% or 1% O_2 for 8 hours, after which whole cell lysates were prepared, and immunoblot assays were performed. (**G**) EVs derived from MDA231-BrM2 subclones were characterized by immunoblot assays. (**H**) EVs derived from 4T1-BR5 cells were stained with antibody against ITGB3, followed by flow cytometry assays.

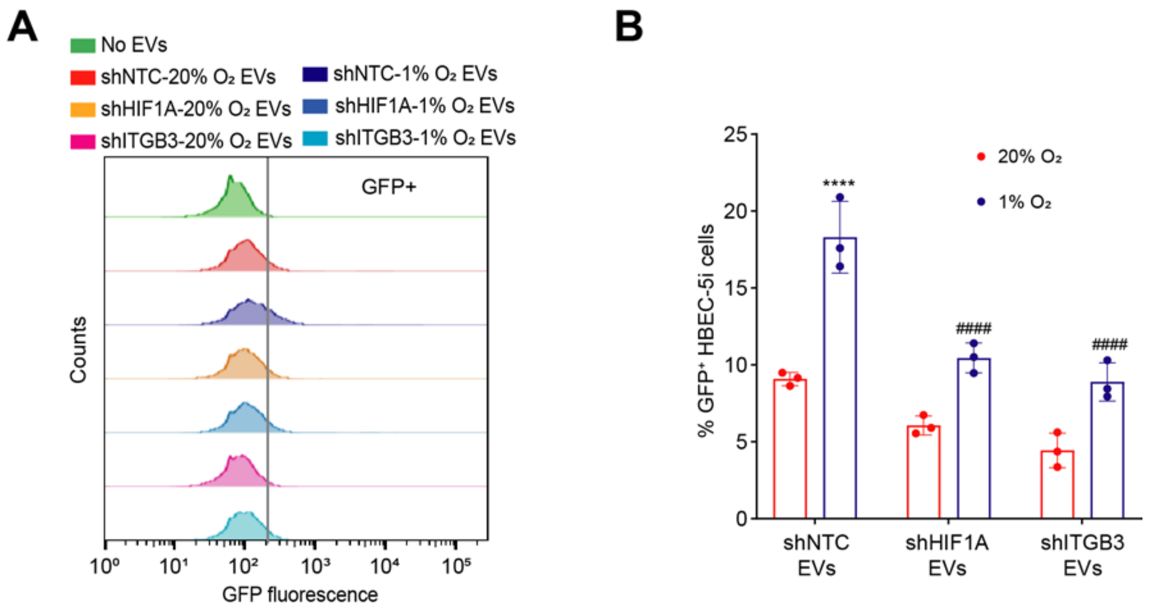

Supplemental Figure 6. HIF-1α and ITGB3 expression promote the interaction of EVs with brain endothelial cells (ECs). (**A** and **B**) GFP$^+$ PalmGRET-EVs (2 µg; derived from MDA231-BrM2 cells exposed 20% or 1% O_2 for 48 hours) were incubated with HBEC-5i ECs for 24 hours, and the cells were analyzed by flow cytometry (**A**) and quantified (**B**; mean ± SD, n = 3). $^{****}P < 0.0001$ versus NTC at 20% O_2; $^{\#\#\#\#}P < 0.0001$ versus NTC at 1% O_2 (two-way ANOVA with Tukey's multiple comparisons test).

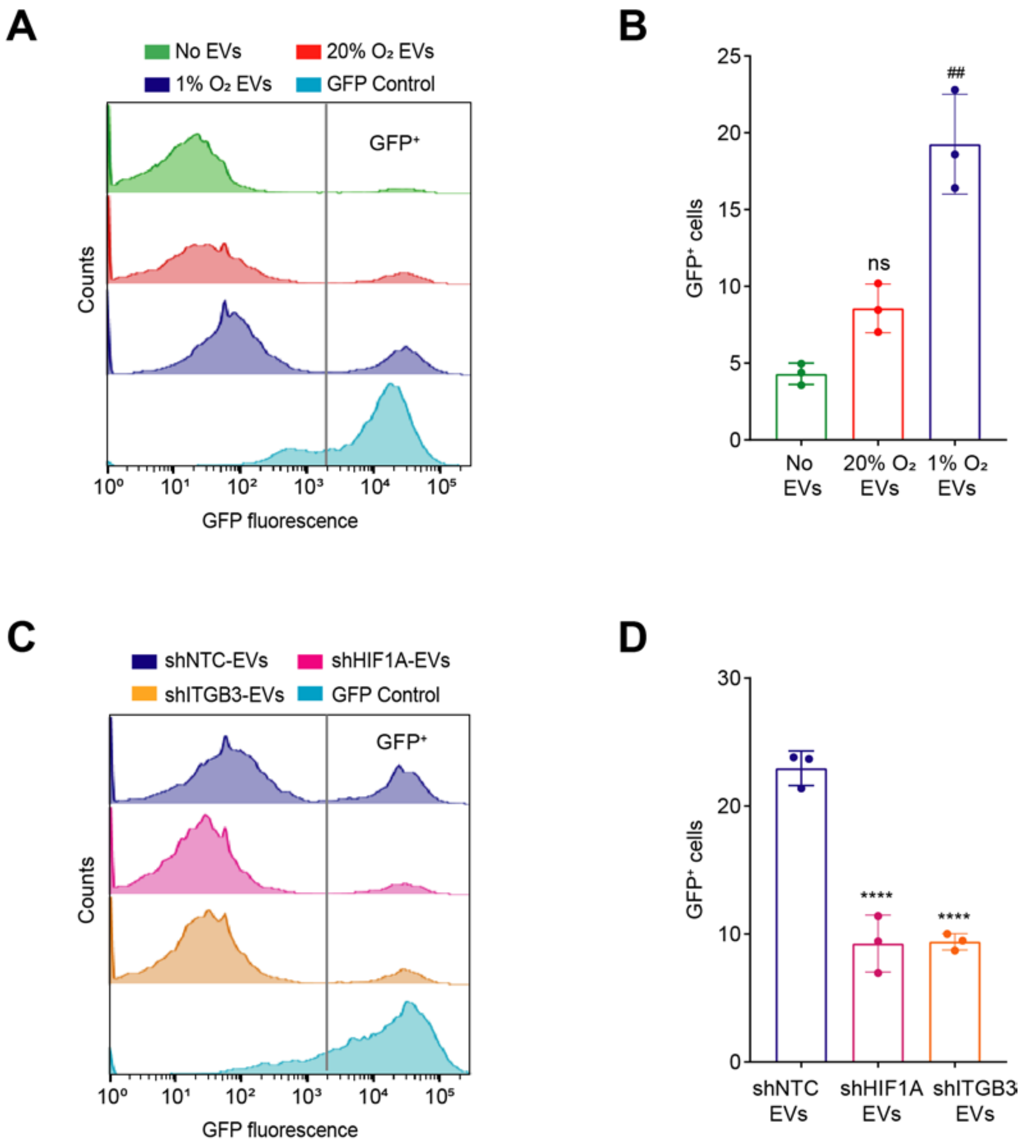

Supplemental Figure 7. ITGB3^{+} EVs promote interaction of BC cells with ECs. (**A-D**) hCMEC/D3 ECs were seeded on 6-well plates, grown to confluency and treated for 24 hours with EVs from MDA231-BrM2 cells that were exposed to 20% or 1% O_2 (**A** and **B**) or subclones that were exposed to 1% O_2 (**C** and **D**). GFP^{+} MDA231-BrM2 cells were then added onto the hCMEC/D3 monolayer, incubated for 1 hour, and non-adherent cells were removed by washing with 1 × PBS. Adherent BC cells were analyzed by flow cytometry (**A** and **C**) and quantified (**B** and **D**; mean ± SD, $n = 3$). $^{**}P < 0.01$ $^{****}P < 0.0001$ versus 20% O_2 EVs or shNTC EVs; ns, not significant versus No EVs (one-way ANOVA with Tukey's multiple comparisons test).

Supplemental Methods

RNA-seq. MDA231-BrM2 subclones were seeded into 6-well plates in three biological replicates and exposed to 20% or 1% O_2 for 24 hours. Total RNA was isolated using TRIzol (Invitrogen, Catalog #1556018) and treated with DNase (ThermoFisher Scientific, Catalog #AM1906). Library preparation and sequencing using the NovaSeq 6000 platform (Illumina) were performed by the Johns Hopkins Genetics Resources Core Facility High-Throughput Sequencing Center. RNA-seq data were processed and interpreted using Genialis Expressions software (https://www.genialis.com). The automated data analysis on the Genialis platform consisted of the following steps: sequence quality checks were performed on raw and trimmed reads (FastQC), trimming and quality filtering of reads (BBDuk), mapping to reference human genome Ensembl v.92 (STAR), expression quantification (featureCounts), and expression normalization (RNA.norm). Key QC metrics (e.g., mapping statistics) were collected. As an additional quality control step, a sample of one million reads (Seqtk tool) was mapped (STAR) separately to human rRNA and globin sequences to evaluate the proportion of these reads in the sample. Differential gene expression analyses were performed with DESeq2. Lowly-expressed genes, which have expression count summed overall samples below 10, were filtered out from the differential expression analysis input matrix. Differential expression results (1% versus 20% O_2) with FDR < 0.05 and mRNA fold change (FC) > 1 were used as cutoff for identification of hypoxia-induced and hypoxia-repressed genes. We also compared different subclones (KD versus NTC) under hypoxic conditions to identify significantly changed mRNA expression (FC > 1 and FDR < 0.05) as HIF-dependent genes. The combined list of mRNAs identified by these two strategies was used for Venn and gene ontology (GO) analysis. Data were analyzed and visualized using Heatmapper for heatmaps, BioVenn for Venn diagrams, and GOnet for GO analysis. Gene set enrichment analysis (GSEA) was performed using GSEA version 2.1.0 software downloaded from the Broad Institute (https://www.gsea-msigdb.org/gsea/index.jsp) using the MSigDB 3.0 gene set signature database following the author's instructions.

ChIP-seq. MDA-MB-231-BrM2 cells were grown on 10-cm adherent culture dishes. At approximately 60% confluency, cells were exposed to 20% or 1% O_2 for 16 hours, fixed in 1% formaldehyde for 10 minutes and quenched with 0.125 M glycine for 5 minutes at room temperature. Cells were washed and collected in 1 × PBS, then split into aliquots of 4 million cells, which were processed using the SimpleChIP Kit (Cell Signaling Technologies) according to the manufacturer's protocol with modifications (97): For each 4-million-cell aliquot, cell membranes were lysed twice in 1 ml of 1 × Cell Lysis Buffer for 10 minutes each, cell nuclei were lysed with 260 μl of 1 × Nuclear Lysis Buffer for 10 minutes then split into two 130-μl Covaris microtubes. Chromatin was sheared using a Covaris E220 sonicator for 9 minutes (cells exposed to 20% O_2) or 12 minutes (cells exposed to 1% O_2) with peak power = 140 watts, duty factor = 5, cycles/burst = 200, and temperature = 4 °C. Aliquots were combined, diluted 5-fold with 1 × ChIP Buffer, and pre-cleared for 30 minutes with 10 μl of magnetic protein A beads. 20 μl of sheared chromatin was isolated to serve as the input for each condition. 2 μg of HIF-1α antibody (Novus Biologicals, Catalog #NB100-479) was added to the remaining chromatin and incubated overnight with rotation at 4 °C. Antibody-protein complexes were bound and precipitated with 30 μl of protein A magnetic beads and washed serially with high salt and low salt buffers according to the manufacturer's protocol. 150 μl of ChIP Elution Buffer was added to each sample. Protein-DNA complexes were reverse crosslinked at 65 °C for 40 minutes, with brief vortexing every 10 minutes. DNA was isolated using a PCR Purification kit (Qiagen). DNA libraries were prepared using the Low Input ChIP-seq kit (Takara Bio) according to manufacturer's instructions. 1 μg of DNA from each sample was amplified with 16 cycles of PCR. Libraries were sequenced at an average depth of 50 million reads using an Illumina NovaSeq sequencer (2 × 100-bp reads).

ChIP-seq data analysis. FastQ files were quality checked and adapter trimmed using Trim-Galore (v0.6.6) with the following flags: --paired --fastqc --clip_R1 10 --clip_R2 10 --three_prime_clip_R1 3 --three_prime_clip_R2 3. Trimmed FastQ files were mapped to the hg19 or hg38 human reference genomes using BWA (v0.7.17), and PCR duplicates marked then removed using Biobambam2 (v2.0.87) and

Samtools (v1.10), respectively. Coverage tracks were generated using the bamCoverage program in the deepTools (v3.5.1) package. ChIP-seq peaks were called using MACS2 in narrow peak mode with input samples as controls, and peaks were overlaid and subsetted using Bedtools (v2.30.0). Heat maps were generated using the deepTools computeMatrix and plotHeatmap commands. Differentially bound peaks were identified using the DiffBind package (v3.8.4) in R (v4.2). Genes with differential HIF-1α binding were identified using Gencode gene annotations.

Transwell migration and invasion assays. Transwell filter chambers (Corning, Catalog #3422), either uncoated or coated with Matrigel (Corning, Catalog #354234), were used for cell migration and invasion assays, respectively. Cells (0.5 and 1.0 × 10^5 for migration and invasion assays, respectively) were seeded in the upper chamber filled with serum-free culture medium, and medium supplemented with 10% FBS was added to the lower chamber. Cells were exposed to 20% or 1% O_2 for 16 hours (migration) or 24 hours (invasion). Cells that had migrated or invaded through the membrane were fixed with paraformaldehyde, permeabilized with methanol, stained with crystal violet and imaged using a light microscope. Quantification was performed in three randomly selected fields per group with Fiji (ImageJ) software. The color images were converted to 8-bit black and white images, and a binary mask was generated by using a threshold of 0-135 with a light background, isolating dark regions corresponding to migrated cells and membrane pores. Non-cellular artifacts, including pores, were removed using the 'Despeckle' function, followed by the 'Remove Outliers' function, in which all bright outliers with radii lower than 30 pixels were excluded. To prevent overcounting, the 'Fill Holes' function was used to remove dark outliers within bright areas. Finally, cells were quantified using the 'Analyze Particles' function, with all continuous bright regions exceeding 1300 $pixel^2$ classified as individual cells. The output was exported as a csv file, containing one row per identified cell with associated area measurements. The number of cells in each image was quantified as the total pixel area of all identified cells (in $pixel^2$). The codes for the quantification of transwell migration and invasion assays are available in GitHub (https://github.com/VarenTalwar/transwell-cellcounting).

NFCM. The NanoAnalyzer (NanoFCM, Inc.) was used to measure the concentration and size of particles in the EV preparations as previously described (98). Two single-photon-counting avalanche photodiodes were used for the simultaneous detection of side scatter and fluorescence of individual particles. The instrument was calibrated separately for concentration and size using 250-nm phycoerythin- and AlexaFluor (AF)488-conjugated phosphatidylserine beads and a Silica Nanosphere Cocktail (NanoFCM), respectively. 20 µl of each EV preparation was incubated with 2 µg of AF488-conjugated ITGB3 or PE-conjugated ITGB4 antibody (Supplemental Table 3) at 37 °C for 30 minutes. After incubation, the mixture was washed twice with 1 × PBS and centrifuged at 100,000 × *g* for 70 minutes at 4 °C. The pellet was resuspended in 50 µl of 1 × PBS. Events were recorded for 1 minute. Using the calibration curve, the flow rate and side scattering intensity were converted into corresponding particle number and size.

NTA. The particles from the EV preparations were diluted in 0.22-µm-filtered 1 × PBS, and a Particle Metrix Zetaview Twin PMX-220 was used to measure the size and concentration of the particles according to the manufacturer's instructions.

TEM. As previously described (98), 10-µl aliquots of EVs were adsorbed to glow-discharged carbon-coated 400 mesh copper grids (Electron Microscopy Science, Catalog #CF400-Cu-UL) by flotation for 2 minutes. Grids were quickly blotted and rinsed by flotation with three drops (for 1 minute each) of Tris-buffered saline. Grids were negatively stained with two consecutive drops of 1% uranyl acetate with tylose in deionized water (passed twice through a 0.22-µm filter), blotted, then quickly aspirated to cover the sample with a thin layer of stain. Grids were imaged on a Hitachi 7600 transmission electron microscope operating at 80 kV with an AMT XR80 CCD (8 megapixel).

In vitro EC permeability assay. Using a kit from MilliporeSigma (Catalog #ECM644), 5×10^4 HBEC-5i or hCMEC/D3 cells were cultured in transwells for 72 hours in complete medium. The cells were treated with EVs or 1 × PBS for 24 hours. Fluorescein isothiocyanate (FITC)-conjugated Dextran (molecular weight ≈ 2000 KDa) was added to the upper chamber. After 20 minutes, fluorescent signals from FITC-

dextran in the lower chambers were captured using a VICTOR Nivo plate reader (PerkinElmer; excitation wavelength = 485 nm; emission wavelength = 530 nm).

Transendothelial migration assay. As previously described (100), transwell inserts with 8-mm pores (Corning, Catalog #3422) were coated with Matrigel (Corning, Catalog #354234) for 4 hours at 37 °C. 5 × 10^4 hCMEC/D3 cells (passages 4–5) were plated on the upper chamber of the inserts, and cells were grown to confluence. One day after confluence, 5 × 10^4 MDA231-BrM2 cells were added to each well. After 24 hours, the cells on the upper side of the insert were removed, then the migrated cells on the lower side were fixed with paraformaldehyde, permeabilized with methanol, stained with crystal violet, photographed, and counted in three random fields per group under a light microscope.

Detection of PalmGRET$^+$ EVs by GFP immunofluorescence. For GFP detection, brain slides were briefly thawed at room temperature before drying at 37 °C for 30 minutes. Tissue sections were then fixed with 4% paraformaldehyde (Sigma-Aldrich, Catalog #P6148) in 1 × Tris-buffered saline (TBS, pH 7.6) for 5 minutes and placed in pre-chilled acetone for 5 minutes. After three washes in 1 × TBS, the sections were blocked in 5% bovine serum albumin (Sigma-Aldrich, Catalog #A9647) and 0.1% Triton X-100 (Sigma-Aldrich, Catalog #T9284) to eliminate non-specific binding prior to incubation with mouse anti-GFP primary antibody (1:1000 dilution; Abgent, Catalog #AM1009a) overnight at 4 °C. Slides were washed three times in 1 × TBS followed by incubation with DyLight488-conjugated goat anti-mouse secondary antibody (1:1000 dilution; Novus Biologicals, Catalog #NB7508G) for 1 hour. The slides were washed three times with 1 × TBS and mounted with Vectashield Antifade Mounting Medium with DAPI (Vector Laboratories, Catalog #H-1200). GFP-labeled coronal sections were examined under an ApoTome.2 fluorescent microscope (Zeiss) with a 40 × oil (Zeiss Immersol™ 518 F, Catalog #10539438) objective lens. Images were captured with an ORCA-Flash4.0 digital camera (Hamamatsu Photonics) and processed using ZEN software (Zeiss). EVs were quantified using ImageJ software (National Institutes of Health). Each fluorescent area datapoint represents the mean of three sections analyzed from a single brain.

Brain section image analysis. Stained mouse brain sections were scanned and visualized with Aperio Imagescope software (v12.4.3, Leica Biosystems, Inc., US). For the analysis of the metastases' area, ImageJ software (v1.8.0, National Institutes of Health) was used. In brief, each tumor metastasis site was manually segmented and automatically calculated with ImageJ. The sum of every metastasis site was expressed as metastasis area (μm^2) and the coronally sectioned brain area was expressed as total brain area (μm^2). The relative brain metastasis area = metastasis area (μm^2)/total brain area (μm^2) × 100%. Three sections per mouse were analyzed and the average of relative brain metastasis area was plotted in the graphs.
