# Supplementary material for: HIF-1 promotes murine breast cancer brain metastasis by increasing production of integrin β3–containing extracellular vesicles
Source: J Clin Invest. 2025 Jul 15;135(14):e190470. doi: 10.1172/JCI190470 (PMC12259260; doi:10.1172/JCI190470)

Full unedited gel for Figure 2

Fig.2B

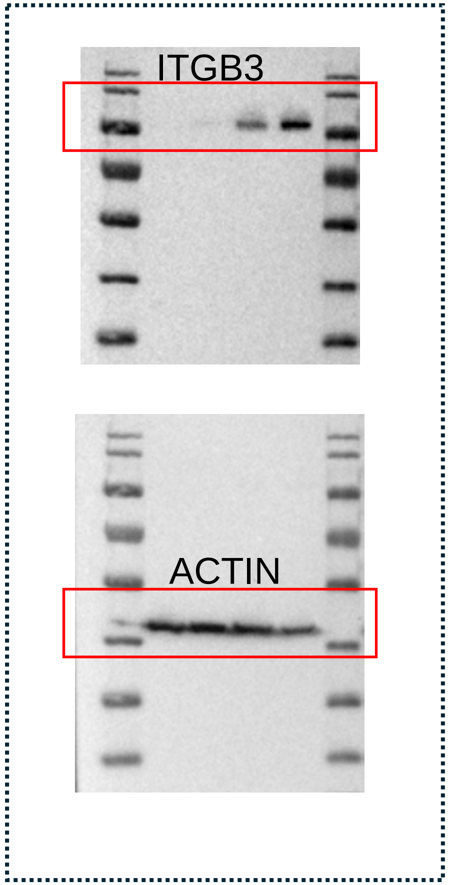

Fig.2D

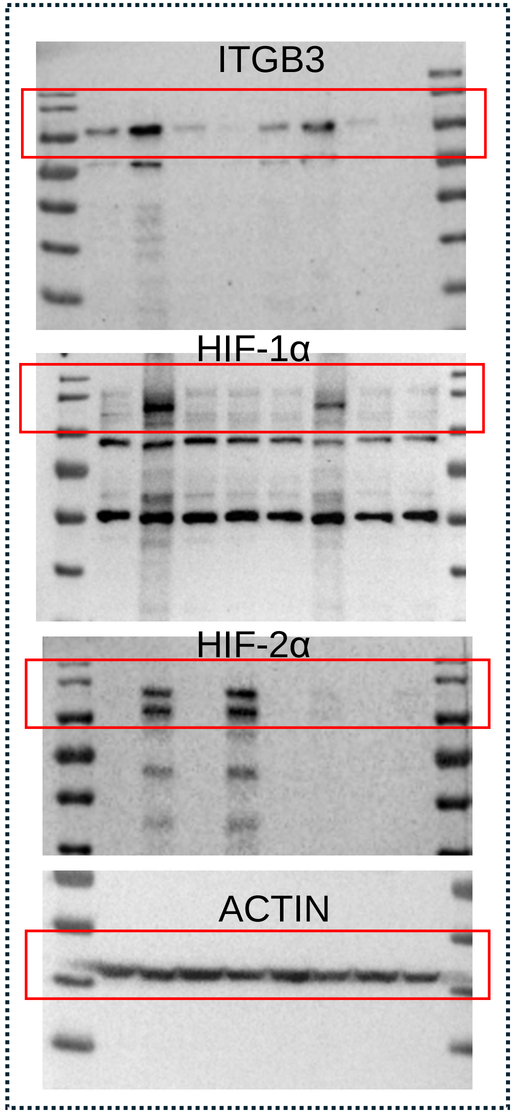

Fig.2F

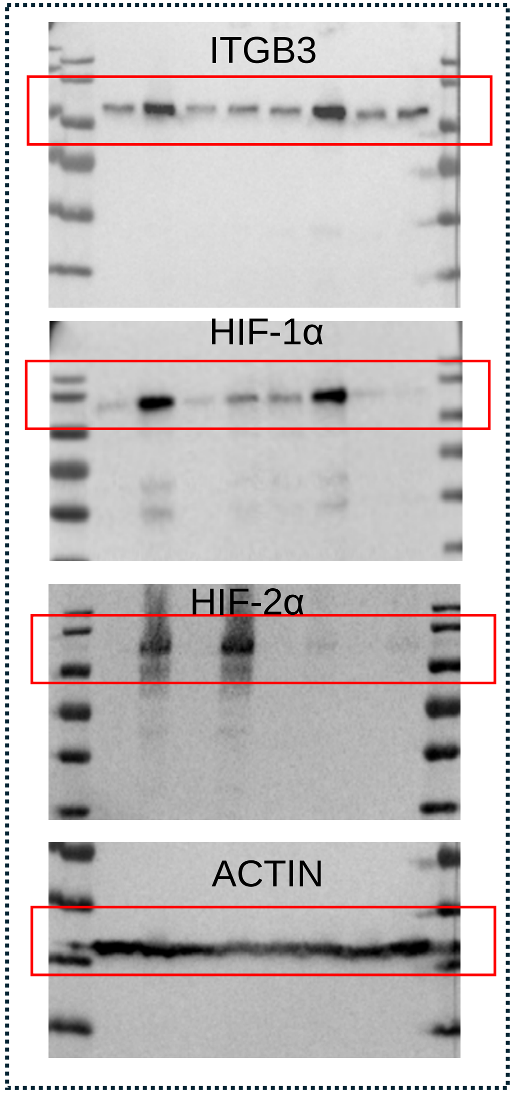

Full unedited gel for Figure 3

Fig.3B

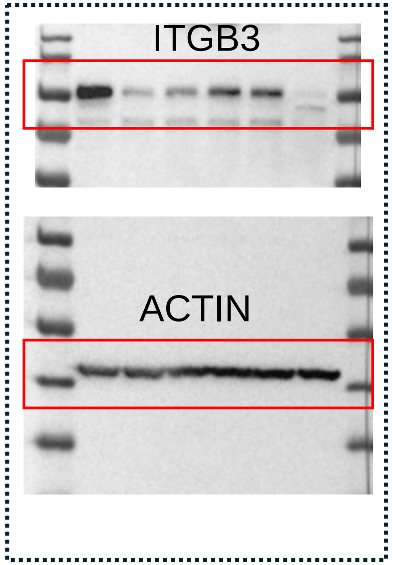

Fig.3D

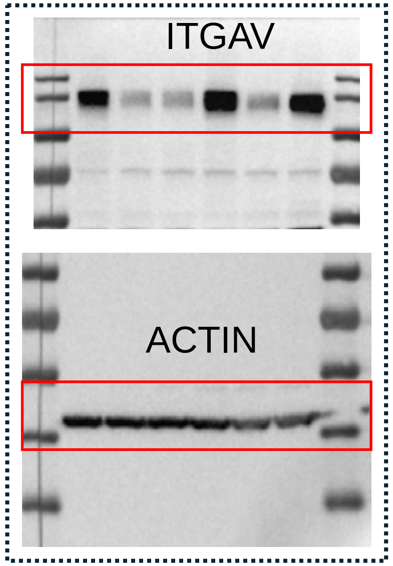

Full unedited gel for Figure 6

Fig.6D

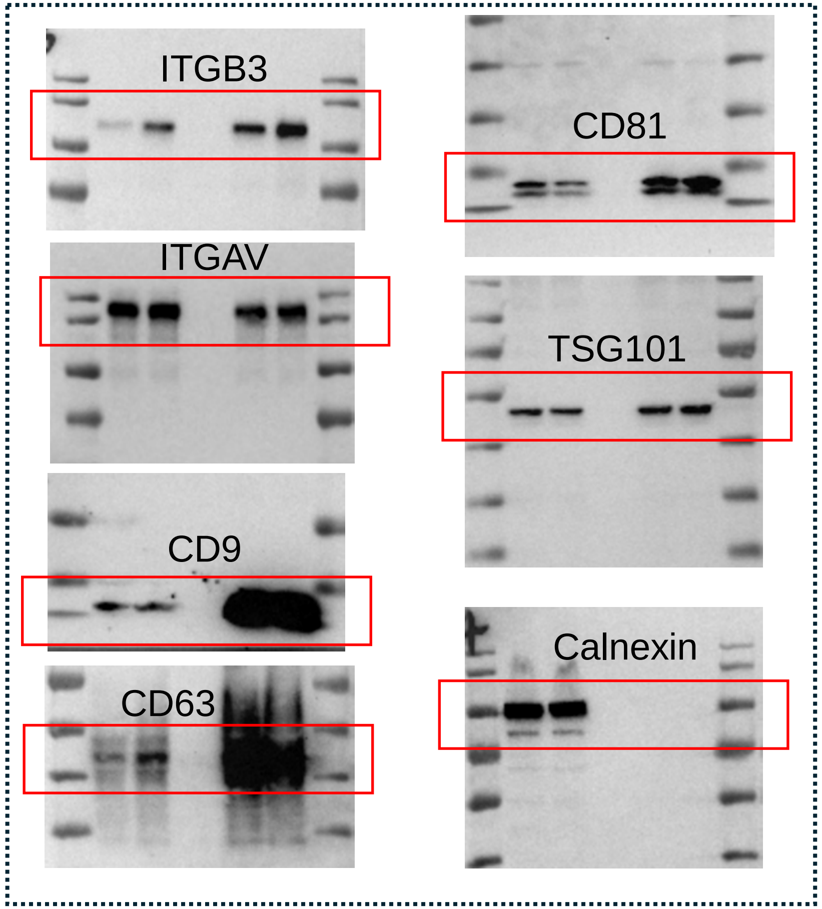

Full unedited gel for Figure 9

Fig.9A

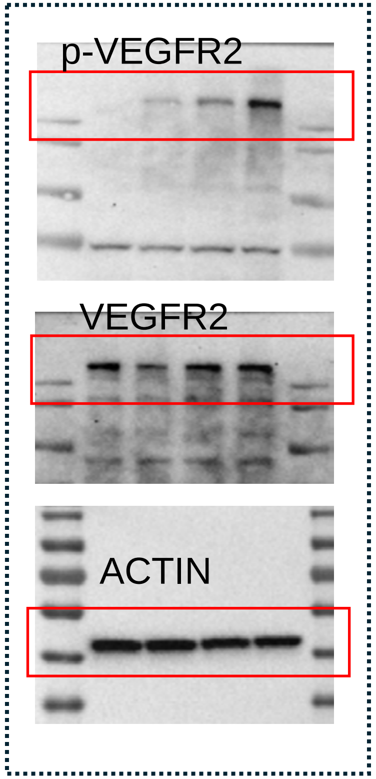

Fig.9B

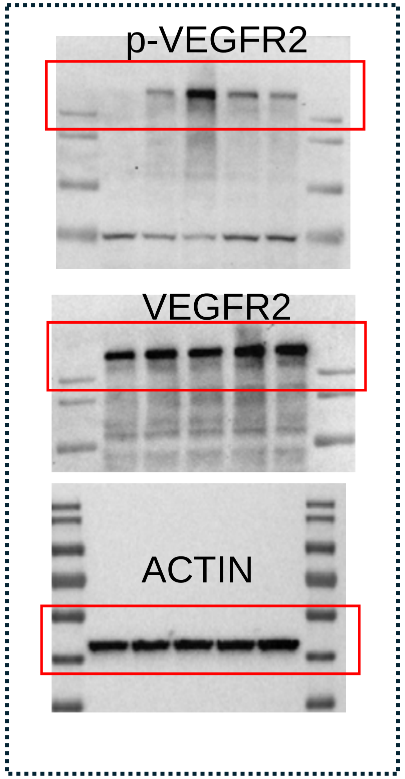

Full unedited gel for Supplemental Figure 2

Fig.S2B

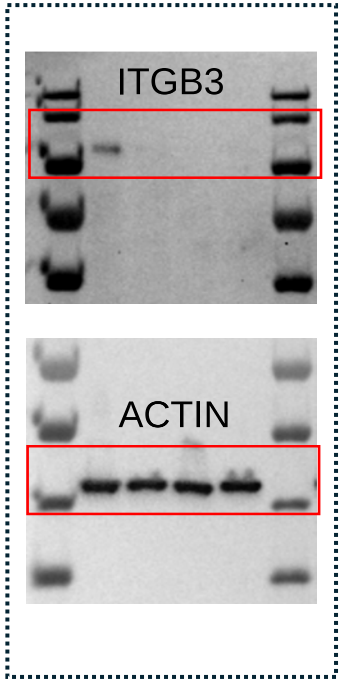

Fig.S2D

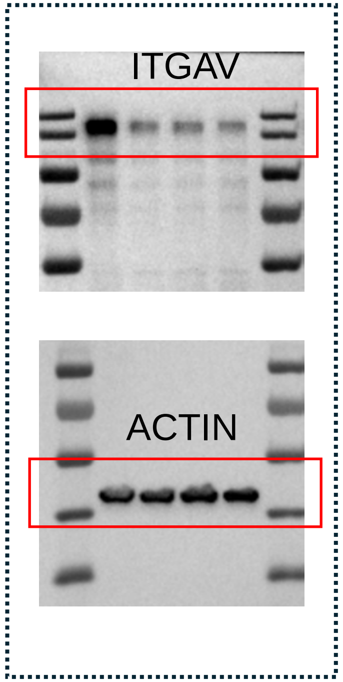

Full unedited gel for Supplemental Figure 3

Fig.S3A

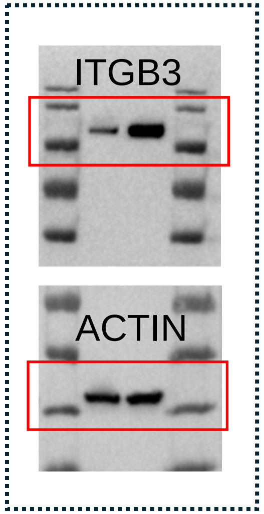

Full unedited gel for Supplemental Figure 4

Fig.S4A

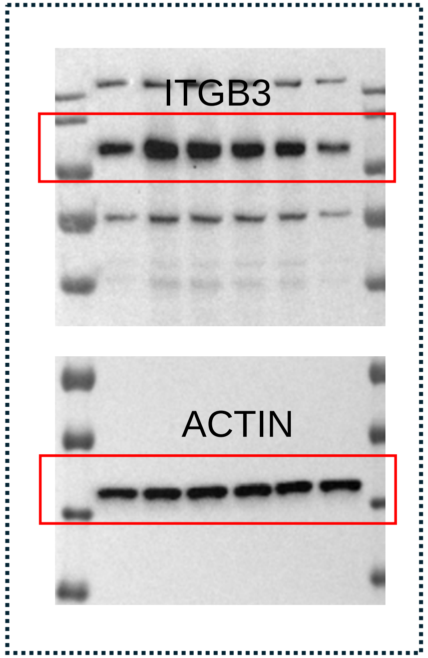

Full unedited gel for Supplemental Figure 5

Fig.S5F

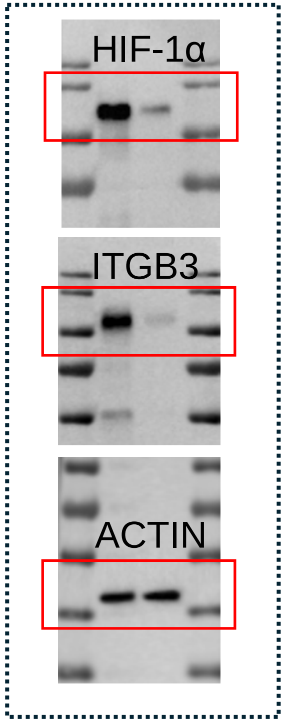

Fig.S5G

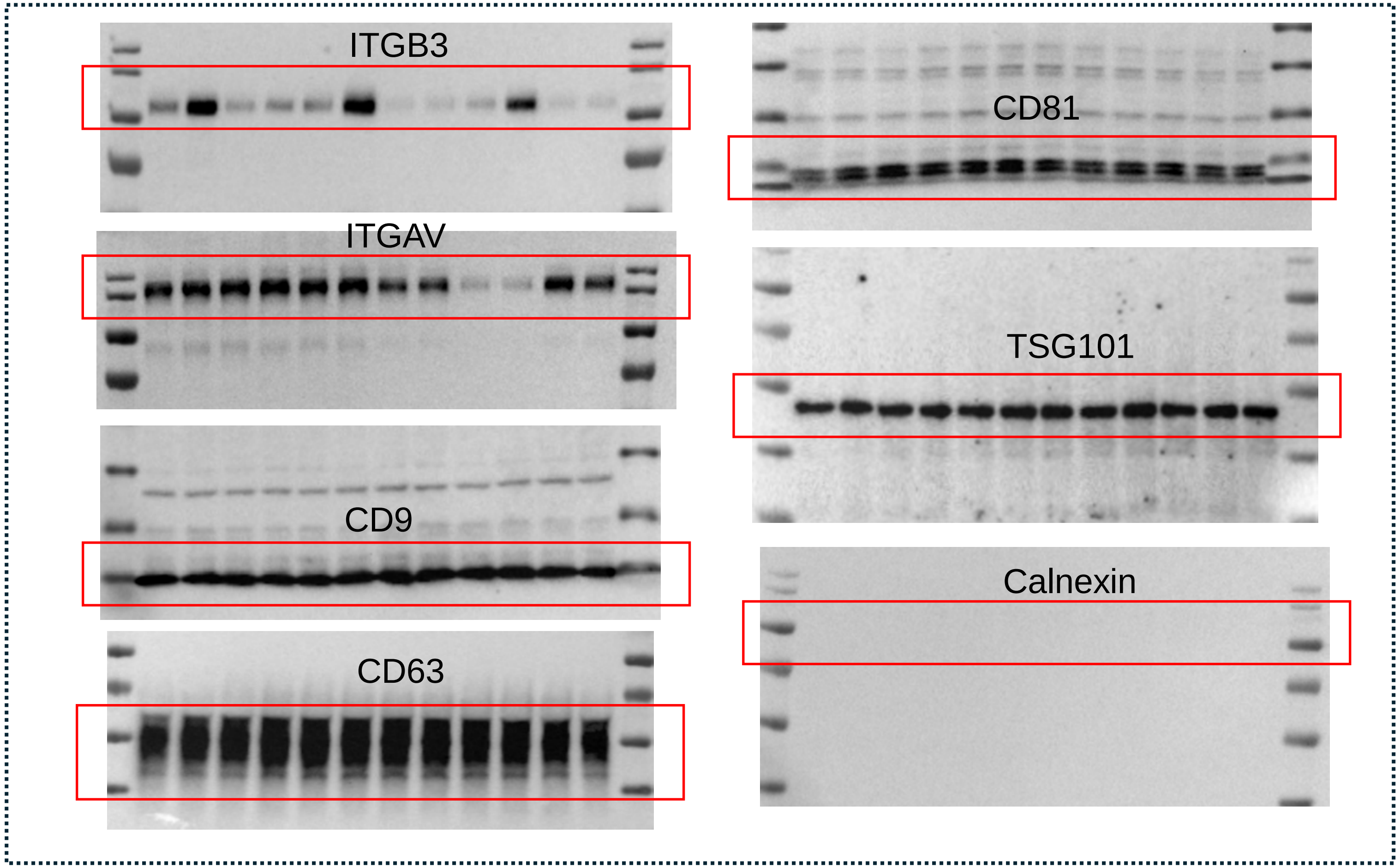

Supplement: Unedited blot and gel images [file jci-135-190470-s150.pdf]
